# The Edge of Stability: Response Times and Delta Oscillations in Balanced Networks

**DOI:** 10.1371/journal.pcbi.1005121

**Published:** 2016-09-30

**Authors:** Grant Gillary, Ernst Niebur

**Affiliations:** 1 Zanvyl Krieger Mind/Brain Institute, Baltimore, Maryland, United States of America; 2 Solomon Snyder Department of Neuroscience, Johns Hopkins University, Baltimore, Maryland, United States of America; Radboud Universiteit Nijmegen, NETHERLANDS

## Abstract

The standard architecture of neocortex is a network with excitation and inhibition in closely maintained balance. These networks respond fast and with high precision to their inputs and they allow selective amplification of patterned signals. The stability of such networks is known to depend on balancing the strengths of positive and negative feedback. We here show that a second condition is required for stability which depends on the relative strengths and time courses of fast (AMPA) and slow (NMDA) currents in the excitatory projections. This condition also determines the response time of the network. We show that networks which respond quickly to an input are necessarily close to an oscillatory instability which resonates in the delta range. This instability explains the existence of neocortical delta oscillations and the emergence of absence epilepsy. Although cortical delta oscillations are a network-level phenomenon, we show that in non-pathological networks, individual neurons receive sufficient information to keep the network in the fast-response regime without sliding into the instability.

## Author Summary

Many networks in the brain are finely balanced, with equal contributions from excitation and inhibition. Deviations from this balance, if for instance the total amount of excitation exceeds that of inhibition, lead to potentially devastating instabilities. Unlike previous work we consider the interaction between fast and slow excitatory connections. We show that not only the amount of excitation needs to be controlled to achieve network stability but also the ratio of slow to fast excitation. Furthermore, optimally fast network performance requires that networks approach instability. However, networks very close to this instability develop oscillations in the delta range (1–4Hz) which potentially cause absence epilepsy. We show that a normal (non-pathological) network can auto-regulate its activity to avoid the instability.

## Introduction

It is generally accepted now that the model of the brain operating as a feedforward system is incorrect. Instead, circuitry in cortex and other brain areas constitutes a finely balanced network of strongly interacting excitatory and inhibitory neuronal populations [1–3]. These networks can respond to their input with high temporal precision [4–6], selectively amplify patterned input signals [7], transmit multiple signals simultaneously between neural assemblies embedded in large networks [8], and maintain activity on a broad range of time constants [9] including those of short-term memory [10]. However, the existence of positive feedback in these networks requires careful maintenance of stability. Previous work [11–13] has shown that stability requires a balance between overall excitatory and inhibitory feedback, see Eq (5) below. Other studies have examined the impact of N-methyl-D-aspartate, (NMDA) on stability in the context of working memory [14, 15] and its effect on homeostasis [16]. However, the impact of fast *α*-amino-3-hydroxy-5-methyl-4-isoxazolepropionic acid, (AMPA) and slow NMDA glutamatergic currents on the relative speed of positive and negative feedback has not been previously examined.

Here, we show that the fact that excitatory currents have two main components with vastly different dynamics requires an additional stability condition, the “temporal balance condition”, which describes the relative speed of positive and negative feedback, Eq (6) below. Even when the strengths of excitatory and inhibitory connections are perfectly balanced, violation of the temporal balance condition makes the network unstable. As we show, deviations from the temporal balance condition by a few percent are sufficient to move the system into a state where it oscillates in the delta range and, subsequently, becomes unstable. This instability may cause absence epilepsy (petit mal) seizures [17–19]. Conversely, as the network approaches this instability it is also able to respond more quickly to changes in input. In fact, we show that near this instability the addition of slow NMDA receptors can make the network respond more quickly than when recurrent feedback is modulated by AMPA receptors alone.

**Fig 1:**
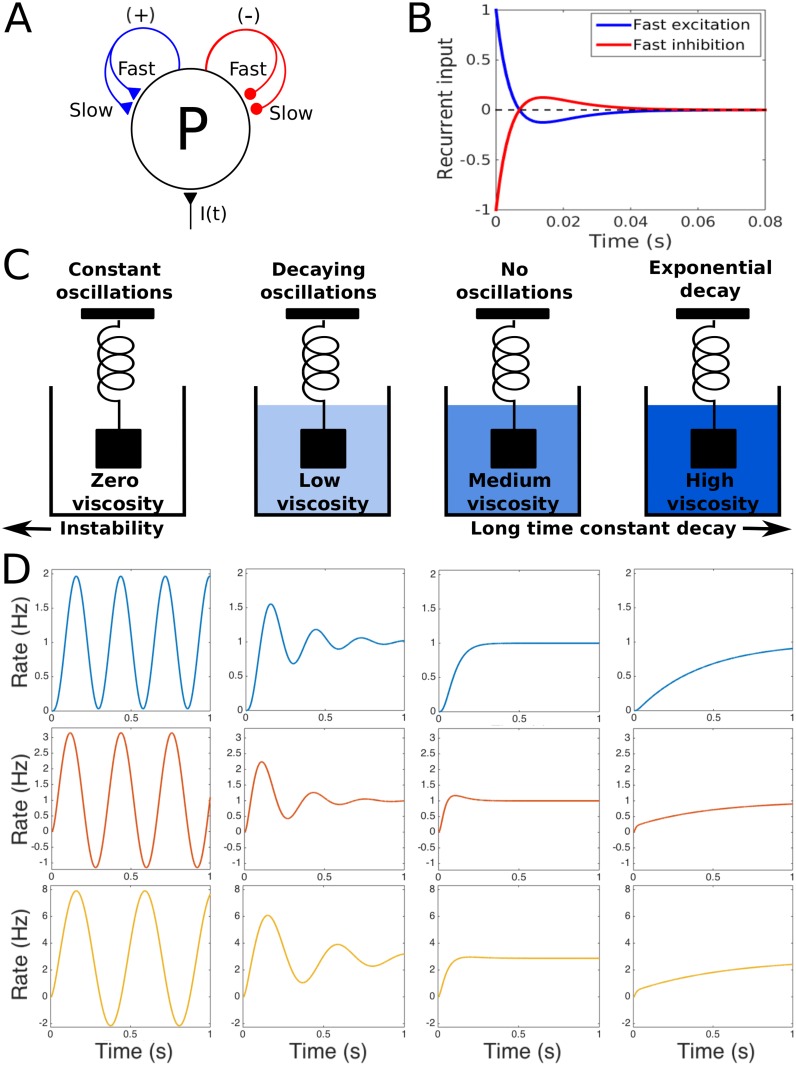
Transient imbalances in the recurrent activity cause the balanced network to act like a damped oscillator. A: Schematic of a population *P* receiving excitatory feedback (+), inhibitory feedback (−), and external input *I*(*t*). Each recurrent projection has a mix of fast and slow receptors, and projections have equal strength on average. B: Response of the recurrent projections in A to an impulse input, *I*(*t*) = *δ*(*t*). If the mix of fast and slow currents in the excitatory connection is biased towards the fast receptors relative to the inhibitory connection then excitation is faster than inhibition and the resulting change in *R* causes a transient increase in input followed by a smaller but longer decrease (blue curve). If inhibition is faster it causes a transient decrease followed by a smaller but longer increase (red). The peak of each response has been normalized to unity. Changes in synaptic strength will scale this response but will not change its shape. We set the fast and slow receptors to be 5 ms and 10 ms in order to allow easy visualization. In all other simulations slow receptors have a time constant of 100 ms unless otherwise noted. C: Schematics indicating the relationship between viscosity for a damped spring and the overall response of the network. D: Response of three systems to a unit step input *I*(*t*) = [1 for *t* > 0; 0 for *t* ≤ 0], as the effective damping constant increases. Top row: Response of the damped harmonic oscillator derived from panel A. Middle: Response of the network shown in A. Bottom: Response of the full network, see Fig 2A.

**Fig 2:**
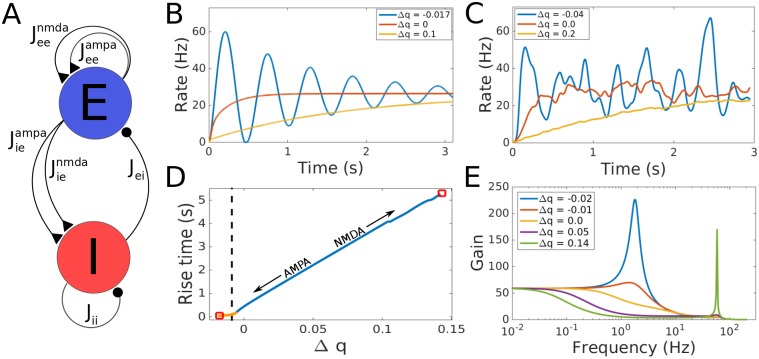
Response of the balanced network to changes in NMDA/AMPA ratio. A: Network schematic showing the structure of the rate model used in simulations. Triangular synapses are excitatory and circular synapses are inhibitory. For LIF networks E and I represent populations of 3,200 and 800 neurons respectively with probability of connection between neurons of *p* = 0.2. B: Simulation of the rate based network for three values of Δ*q*. At the smallest value, delta oscillations appear (blue line). This value is in the orange range in D, for even smaller values the system is unstable. All rate based networks use *k* = 1.2, *w* = 30. C: Same for the LIF network but with *k* = 0.65 and *w* = 5.0. D: Rise time in seconds as a function of Δ*q* for the rate model. Red squares indicate instabilities, the orange segment represents the values of Δ*q* which generate delta oscillations, and the dashed black line is at the value of Δ*q* where the rise time is 100 ms. E: Frequency response of the linear system. Delta oscillations start for small negative Δ*q* (blue) and gamma oscillations (green) appear when Δ*q* approaches the right instability in D.

**Fig 3:**
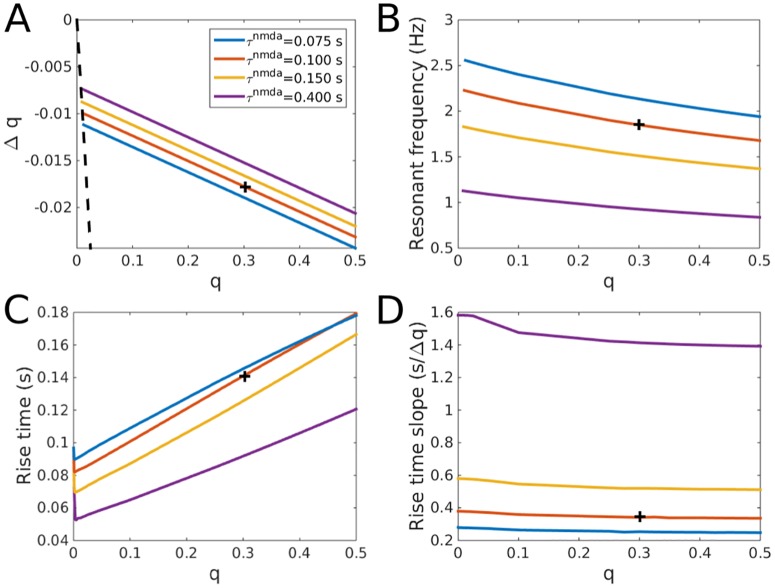
Dependence of stability, delta oscillations and rise time on NMDA receptors. A: Values of Δ*q* for which the network in Fig 2A is unstable as a function of *q* and *τ*^*nmda*^. The dashed black line indicates the maximum absolute value of Δ*q* possible for each corresponding value of *q*. The intersections between the dashed black line and colored lines indicate the values of *q* below which each network is stable for all possible values of Δ*q*. B: The resonant frequency for each oscillatory instability in panel A. C: The fastest non-oscillatory (critically damped) rise time for each network as a function of *q* and *τ*^*nmda*^. The rise time was computed at the value of Δ*q* where delta oscillations appear or, when *q* is too small to allow for the emergence of delta oscillations, at the most negative possible value of Δ*q*. D: The slope of the rise time in the overdamped parameter regime for each 0.01 change in Δ*q*, see the slope of the line in Fig 2D where Δ*q* > 0. The slope was computed from the rise time values for Δ*q* between 0 and 90% of the value of Δ*q* at the gamma oscillatory instability. All networks used *w* = 30 and *k* = 1.2. Black crosses indicate values of *q* and *τ*^*nmda*^ for network in Fig 2B–2E.

**Fig 4:**
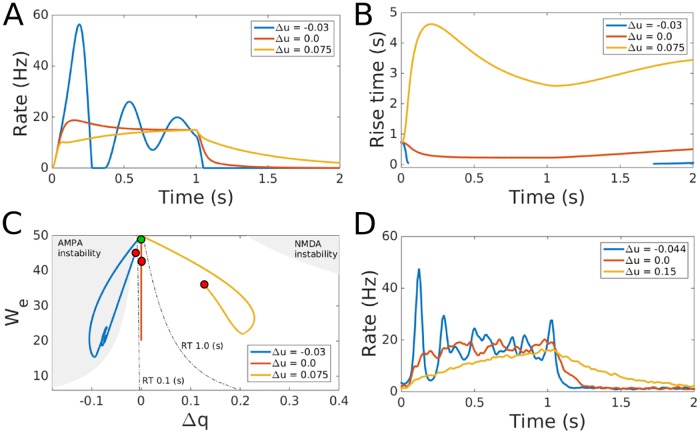
Dynamics of the balanced network with STD and different usage rates for NMDA and AMPA in the EE projections. Positive Δ*u* means higher usage rate for NMDA than AMPA synapses. A: Response of the rate based network with STD to a square pulse imput, beginning at *t* = 0 and ending at *t* = 1 s. Parameters are *k* = 1.2, *w* = 50, *q* = 0.5, *u* = 0.2 and *τ*_*r*_ = 500 ms. B: Rise time of linear networks for the parameters computed from the time dependent synaptic strengths in panel A using the *risetime* function from Matlab (The MathWorks, Inc., Natick, MA). For Δ*u* = −0.03, the system is unstable where the blue trace is not shown. C: Temporal trajectories of the network simulated in A as a function of $W_{e}=J_{ee}^{ampa}+J_{ee}^{nmda}=\left(J_{ie}^{ampa}+J_{ie}^{nmda}\right)/k$ and Δ*q*. The green circle indicates the starting point of the trajectories while the red circles indicate the end points of the trajectories. The parameters were computed from the STD modulated synaptic strengths at each time point. Shaded areas indicate where the linear system is unstable for the same set of parameters. The AMPA instability corresponds to the left red square in Fig 2D and the NMDA instability to the right red square. Dashed lines indicate parameters where the linear networks have rise times (RTs) of 0.1 s and 1.0 s. D: Response of the LIF network with STD to a square pulse beginning at *t* = 0 and ending at *t* = 1 s. Parameter values are *k* = 0.65, *w* = 10, *q* = 0.5, *u* = 0.2 and *τ*_*r*_ = 1,000 ms.

To understand stability and response times in networks with AMPA and NMDA projections, we will study populations with a balance of excitatory and inhibitory input. To gain an intuitive understanding of such a network, we will begin with a simplified single population having both excitatory and inhibitory recurrent connections, Fig 1A, whose basic dynamics are those of a simple second-order system (dampened spring), Fig 1C. We then examine a network with both excitatory and inhibitory populations, Fig 2A, and describe how the long time constant NMDA receptors impact network oscillations and response times, Fig 3. Finally, we show that the described behavior is naturally produced in networks with short-term depression (STD), Fig 4, and that it occurs not only in mean-rate approximations but also in spiking networks, Figs 2C and 4D.

**Fig 5:**
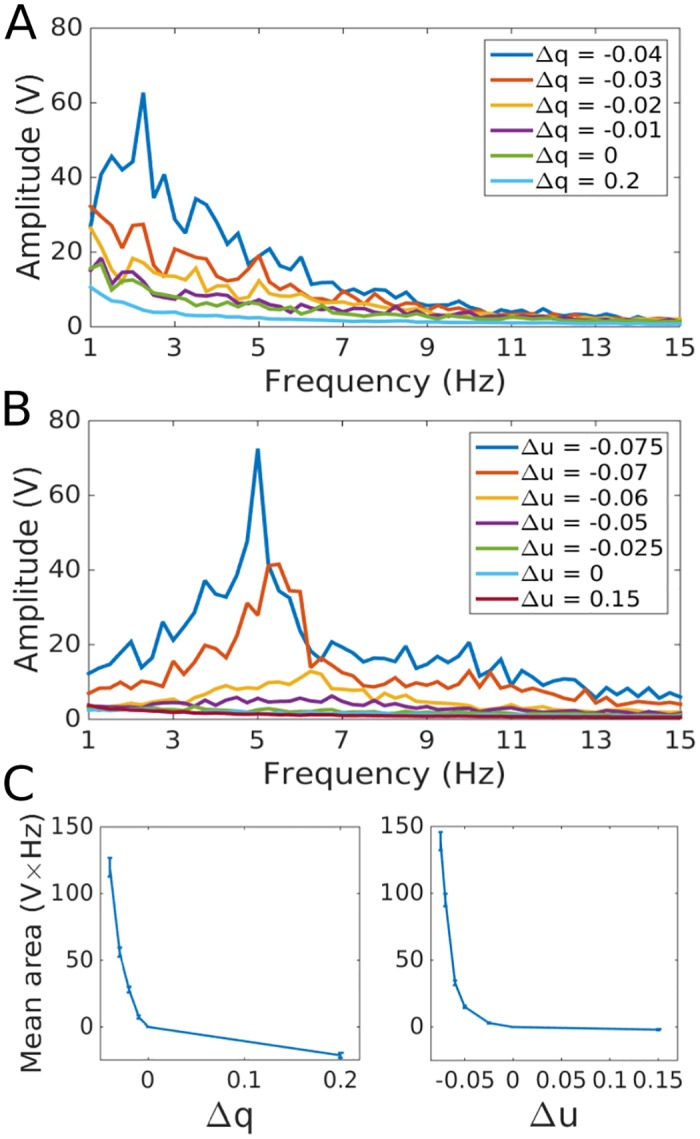
Frequency responses of the LIF network to constant Poisson input. Network parameters are as in Figs 2C and 4D. A: Average over ten runs of the frequency response for each excitatory neuron in the LIF network without STD for different values of Δ*q*. B: Same as A but with STD. The independent variable is Δ*u* rather than Δ*q*. C: Mean of the area under each LIF neuron’s frequency response between 0.5 and 5.5 Hz for the LIF network without STD (left) and between 3.0 and 8.0 Hz for the LIF network with STD (right). The mean value of the integral for Δ*q* = 0 and Δ*u* = 0 for each neuron was subtracted for all data points. Error bars are standard deviations.

Since, as we show, small changes in the AMPA/NMDA ratio can affect both a network’s stability and its response time, homeostatic mechanisms that maintain the balance of the time constants of EE and IE projections are required to keep the system stable and, at the same time, its dynamics in the physiological range. Current theories of homeostasis use as control parameter either the average firing rate of a neuron or the activity history of individual synapses, and corrections are implemented in terms of spike timing dependent plasticity mechanisms [20]. Neither of these provides the information needed for maintaining the AMPA/NMDA ratio within the stability range of the temporal balance condition. What is needed is, instead, information about the frequency response of the network. We show that individual neurons in a spiking network have sufficient information about the network frequency response to allow them to identify, and if necessary counteract, instability in the local network, Fig 5. Failures of homeostatic control may allow the system to transition through the oscillatory regime into the instability, leading to absence epilepsy.

## Results

### Transient Imbalances and the Damped Harmonic Oscillator

A network is considered to be balanced if its excitatory and inhibitory inputs cancel in the long term, even though transient changes can still have a significant impact. Fig 1A shows a network with a single population, *P*. This network has one excitatory and one inhibitory recurrent projection, each with a combination of fast and slow receptors, and both having exactly the same strength over the long term. Therefore, if the ratios of fast to slow connections are equal on both projections, excitatory and inhibitory feedback exactly cancel at all points in time. However, if the ratio of fast to slow inputs on the excitatory projection is increased compared to the inhibitory projection, small increases in the firing rate of the population will cause a transient excess of excitatory feedback followed by a smaller but longer period of inhibition, Fig 1B. On the other hand, when the ratio of fast to slow inputs on the inhibitory feedback is greater, a small increase in firing rate will cause a transient inhibitory feedback followed by a smaller but longer period of excitation. Since the overall strength of the connections are the same, the total recurrent excitation and inhibition still ends up balanced. These transient but balanced changes act mathematically like a first derivative which either slows down or speeds up the network, similar to the “derivative networks” that have been previously described [10]. For physical systems the first derivative often acts like a friction term, or viscosity in a fluid. The network in Fig 1A can be approximated by a spring in a viscous fluid with the equation,

$$\frac{d^{2}R\left(t\right)}{dt^{2}}+2\zeta \omega_{0}\frac{dR(t)}{dt}+\omega_{0}^{2}R\left(t\right)=I\left(t\right).$$ (1)

where *R*(*t*) is the firing rate of the population *P*, *ζ* is a damping coefficient, *ω*_0_ is the frequency at which the spring oscillates when *ζ* = 0, and *I*(*t*) is some time dependent input. A full derivation of this representation, showing how it is approximated from the balanced network described in Eqs (2)–(4) and shown in Fig 2A is given in the Supplement. In the spring approximation, changes in the relative timing of excitatory and inhibitory feedback change the damping coefficient, *ζ*, effectively acting like changes in the viscosity of a fluid surrounding the spring.

The diagrams in Fig 1C depict four different types of dynamical responses that a spring can exhibit as a function of *ζ*. The first type of response is where the fluid has no impact at all. In this case the spring oscillates with a constant amplitude and at the system’s natural frequency *ω*_0_, about 3.5 Hz for realistic cell and synaptic parameters in the spring approximation where $\omega_{0}=1/\sqrt{\tau_{e}\tau^{nmda}}$. We note that the units of *ω*_0_ in this formula are rad/s. We define *τ*_*e*_ as the membrane time constant and *τ*^*nmda*^ as the decay time of the NMDA receptors (see Supplement). This behavior, shown in the first column in Fig 1D, is called an undamped response. The increasing frequency of *ω*_0_ that can be observed as the full system is reduced to the harmonic oscillator, Fig 1D bottom row compared to the top row, is due to a reduction of the number of time constants in the system. For example, setting the value of *τ*^*ampa*^ = 0 removes two terms in the denominator of the equation for *ω*_0_, compare the analytical solutions in Eqs (S5) and (S11), causing a commensurate increase in *ω*_0_. Although we do not have an analytical solution for the full network, the addition of *τ*_*i*_ and *τ*^*gaba*^, where GABA is gamma-Aminobutyric acid, could explain the lower value of *ω*_0_ for the full network. For even smaller values, *i.e.* *ζ* < 0, the network is unstable and oscillations will increase in an unbounded manner. On the other hand, for increased viscosity a step input generates a transient oscillation which decays over time toward constant steady state activity, second column in Fig 1C and 1D. This is an underdamped system, 0 < *ζ* < 1. As the viscosity continues to increase it reaches a point where oscillations are no longer possible and the system responds with an exponential decay to the steady state, third column in Fig 1C and 1D. This system is called critically damped, *ζ* = 1. A critically damped response is the fastest possible non-oscillating response for that system. Any further increases in viscosity continue to slow the exponential approach of the network of its steady state, fourth column in Fig 1C and 1D. Such networks are called overdamped, *ζ* > 1.

So far we have discussed the behavior of the idealized spring system, Eq (1), shown in the top row of Fig 1D. The middle and bottom rows show, respectively, the equivalent results for the reduced network depicted in Fig 1A, and the full network, Fig 2A. Clearly, the spring model approximates well the responses of both of these networks. The behavioral repertoire of all three systems comprises an unstable regime, undamped and damped oscillations (first and second column), fast responses to input close to critical damping (third column), and a slower asymptotic approach to steady state for larger damping (fourth column). For both network models (Figs 1A and 2A), these different behaviors are obtained by only changing the ratio of fast and slow inputs on the excitatory-to-excitatory (EE) or excitatory-to-inhibitory (IE) projections. In the next section we will consider in more detail how this activity comes about in the full network of Fig 2.

### Rise Time and Stability in the Linear Network

We now consider a recurrent rate-based network with AMPA, NMDA and GABA synapses, shown schematically in Fig 2A and described by

$$\begin{array}{ll} \tau_{e}\frac{dR_{e}}{dt}= & -R_{e}+J_{ee}^{ampa}S_{ee}^{ampa}+J_{ee}^{nmda}S_{ee}^{nmda}\\ & -J_{ei}^{gaba}S_{ei}^{gaba}+I\left(t\right) \end{array}$$ (2)

$$\begin{array}{ll} \tau_{i}\frac{dR_{i}}{dt}= & -R_{i}+J_{ie}^{ampa}S_{ie}^{ampa}+J_{ie}^{nmda}S_{ie}^{nmda}\\ & -J_{ii}^{gaba}S_{ii}^{gaba} \end{array}$$ (3)

$$\tau_{mn}^{l}\frac{dS_{mn}^{l}}{dt}=-S_{mn}^{l}+R_{n}$$ (4)

Variables *R*_*e*_ and *R*_*i*_ represent the firing rates of the excitatory and inhibitory populations, with intrinsic time constants *τ*_*e*_ and *τ*_*i*_. $J_{mn}^{l}$ is the synaptic strength of the projection from population *n* to population *m* of synaptic type *l* which is GABA for the inhibitory and either AMPA or NMDA for the excitatory projections. $S_{mn}^{l}$ is the synaptic activation level of the projection from population *n* to population *m*, with synaptic time constant $\tau_{mn}^{l}$. Time varying input to the excitatory population is denoted by *I*(*t*).

We use two primary concepts to describe the speed at which a network responds to inputs: network time constant and rise time. We use the term network time constant, *τ*_*n*_, to represent the time constant associated with the dominant eigenvalue, *λ*, of the network where *τ*_*n*_ = −1/*λ*. However, in many cases dominant eigenvalue approximations are not appropriate. Therefore, in order to compare across all networks, we also use the term rise time which is the time it takes the network to go from 10% to 90% of its steady state value. When the dominant eigenvalue approximation is appropriate, consider a system described by a single decaying exponential with time constant *τ*_*n*_, then the rise time is approximately equal to ln(9) × *τ*_*n*_.

Projections from the excitatory population have AMPA and NMDA components, each carrying part of the total synaptic strength. The stability conditions and time constants for the linear network can be derived from its eigenvalues, all of which need to have negative real parts for the system to be stable. Assuming that the synaptic strength *J* is large compared to the neuronal leak current and that all projections are *O*(*J*), we develop approximate conditions for both stability and the network time constant, *τ*_*n*_ by approximating the coefficients of the characteristic polynomial in the highest order of *J*. Requiring the coefficients of the characteristic polynomial to be all positive is a necessary condition for stability while the ratio of the first two coefficients determines *τ*_*n*_. This leads to two conditions for the network which are derived in the supplementary material,

$$J_{ei}\left(J_{ie}^{ampa}+J_{ie}^{nmda}\right)-J_{ii}\left(J_{ee}^{ampa}+J_{ee}^{nmda}\right)>0$$ (5)

$$\begin{array}{cc} J_{ei}(J_{ie}^{ampa}\tau_{ie}^{nmda}+ & J_{ie}^{nmda}\tau_{ie}^{ampa})\\ - & J_{ii}\left(J_{ee}^{ampa}\tau_{ee}^{nmda}+J_{ee}^{nmda}\tau_{ee}^{ampa}\right)>0 \end{array}$$ (6)

For ease of notation, we omit the superscript *gaba* for the inhibitory connections in this equation.

In both equations, the first term characterizes negative feedback and the second positive feedback. Eq (5) is the previously described condition for the balance between strengths of inhibitory and excitatory connections [10, 21]. When the left-hand side (LHS) of this “balance condition” equation becomes negative, the network is unstable since recurrent negative feedback (the first term) is smaller than recurrent positive feedback (second term). If the LHS is positive, the network is stable and dominated by inhibition. When Eq (5) is fulfilled, the novel “temporal balance condition,” Eq (6), describes instabilities due to the relative *timing* of negative and positive feedback. If negative feedback weighted by synaptic strength (first term) is too slow relative to the weighted positive feedback (second term), *i.e.* when the temporal balance condition becomes negative, it cannot balance the excitatory feedback, thus making the network unstable. This is true even if total negative feedback (first term in Eq (5)) is stronger than the total positive feedback (second term in Eq (5)), *i.e.* when the steady state network is dominated by inhibition. On the other hand, positive and increasing values of the LHS of Eq (6) lead to increases in *τ*_*n*_ which can vary over a large range. When the LHS of Eq (6) approaches *O*(*J*^2^), then *τ*_*n*_ increases up to the range of several seconds. The network is then similar to the negative derivative feedback network introduced as a model for working memory [10]. While that study defined the stability conditions in terms of a single time constant on both excitatory projections, we focus on the more biophysically realistic implementation in terms of AMPA and NMDA receptors. Since the proportion of AMPA and NMDA receptors on each projection controls both the value of *τ*_*n*_ and the stability of the network there is a direct trade-off between speed of input response and stability. When the network responds quickly it is close to an AMPA dominated instability. As the NMDA component increases, the network moves further from this instability and *τ*_*n*_ increases.

In order to analyze the stability of this network a change of parameters is useful. Let *w* be the base synaptic strength, *k* the inhibitory to excitatory ratio, *q* the proportion of synaptic strength carried by NMDA receptors (on all excitatory projections), and Δ*q* a relative shift in the proportion of synaptic strength through NMDA and AMPA receptors on the EE projections only. With *q* ∈ [0, 1] and Δ*q* ∈ [−*q*, 1 − *q*], we have $J_{ee}^{ampa}=\left(1-q-\Delta q\right)w$, $J_{ee}^{nmda}=\left(q+\Delta q\right)w$, $J_{ie}^{ampa}=\left(1-q\right)w$, $J_{ie}^{nmda}=qw$ and $J_{ii}^{gaba}=J_{ei}^{gaba}=kw$. Clearly, *k* parametrizes the impact of inhibitory *vs.* excitatory synapses and *q* the relative strength of NMDA *vs.* AMPA. Varying Δ*q* represents changes in the temporal balance, note that for this parameterization Eq (6) is zero when Δ*q* = 0. We chose to modulate the relative difference in time constants between EE and IE projections by adding Δ*q* to EE; subtracting Δ*q* from IE yields the same network dynamics.

The simulation in Fig 2B shows the response of a linear network with *q* = 0.3, *k* = 1.2 and *w* = 30 to a step input at *t* = 0. Instability occurs for relatively small deviations from temporal balance. Increasing the AMPA contribution by setting Δ*q* = −0.02 makes the network unstable. The network resonates with a low frequency (in the delta range) as it approaches instability. S1F and S1G Fig shows the resonant frequency due to the AMPA dominated instability as a function of *w* and *k*. The frequency is fairly robust, varying between 1.4 Hz and 2.8 Hz over a large range of parameter values. The network is, however, very sensitive to changes in Δ*q*: increasing Δ*q* by 0.1 towards NMDA currents increases the rise time of the network to approximately 5 seconds. For even larger Δ*q* (slightly below 0.15) another instability occurs, to be discussed below.

A similar set of behaviors occurs for networks of leaky integrate and fire (LIF) neurons. Fig 2C shows the response of a network of 3,200 excitatory and 800 inhibitory neurons to a step input at *t* = 0. In our simulations, each LIF neuron has a connection probability of *p* = 0.2 to all other neurons. The average synaptic strength from each population onto a downstream neuron is *w* = 5.0, and the relative strength of inhibition is *k* = 0.65. Results are qualitatively similar to those of the rate model, Fig 2B. However, there is an increase in the (absolute) values of Δ*q* at which the network approaches either one of the instabilities (compare figure legends). The overall behavior is the same as in the rate network, a transition from instability and low frequency (delta) oscillations for negative Δ*q* to long time constant integration for large and positive Δ*q*.

Fig 2D illustrates the trade-off between a network’s stability and its rise time for a step input. For the network to respond quickly to an input, it must maintain small negative values of Δ*q* close to the dashed black line representing a rise time of 100 ms. Any increase in Δ*q* will cause a large concurrent increase in *τ*_*n*_ slowing down the network response. Conversely, decreasing Δ*q* leads to the emergence of a bifurcation with poles in the transfer function which move away from the real axis (S1H Fig). The right edge of the orange portion of the line in Fig 2D represents this bifurcation. As Δ*q* becomes more negative, the resulting oscillations in the delta range (1–4 Hz, blue lines in Fig 2B, 2C and 2E) continue to increase in frequency until the poles cross the imaginary axis (S1H Fig) and the network becomes unstable, left red square in Fig 2D. To maintain fast response times, the system has to stay close to this instability. Therefore, balanced networks with short response times sit on the edge of stability: relatively small uncompensated changes in AMPA strength on the EE projections yield either an unstable network or a much slower stimulus response, both highly undesirable in sensory cortex.

For large Δ*q* the network rise time increases beyond 5 seconds at which point gamma-range oscillations appear (Fig 2E, green). Subsequently, the poles of the transfer function cross the imaginary axis, S1H Fig, and the system becomes unstable, right red square in Fig 2D. Thus, our model predicts that increasing *τ*_*n*_ by increasing the proportion of NMDA over AMPA receptors results in gamma oscillations and eventual instability. This type of gamma oscillations has been described previously [22]. The rise times spanned in Fig 2D extend all the way from physiologically realistic responses to sensory stimuli (tens of ms) to persistent activity suitable for working memory (seconds). These large shifts in the response dynamics of the network occur for a change in Δ*q* less than 0.2.

The emergence of delta oscillations in balanced networks is consistent with the Stargazer model of absence epilepsy. Stargazer mice lack the stargazin protein which is expressed in inhibitory interneurons and is involved in AMPA receptor trafficking [17–19]. Animals lacking this protein are prone to seizures with increased EEG power in the delta and low theta range. The cause of these seizures has previously been ascribed to a reduction in the strength of inhibitory feedback from the loss of AMPA receptors on inhibitory interneurons [19, 23] but this does not explain the occurrence of delta oscillations. While decreased inhibitory feedback may also play a role, our model suggests that a relative slowing of the IE projection through a reduction in the proportion of AMPA receptors breaks the temporal balance condition. Since, as discussed, stability depends on the *relative* timing of the EE and IE projections rather than their absolute values, a reduction of AMPA in the IE projection is equivalent to an increase in AMPA on the EE projection and will also induce a delta oscillatory instability.

This view is strongly supported by recent work showing a compensatory mechanism which increases the strength of NMDA currents in stargazin deficient mice [18]. The addition of NMDA receptors compensates for the disinhibitory effect caused by the loss of AMPA receptors, allowing the system to fulfill the balance condition, Eq (5). However, the increase of NMDA also slows the negative feedback which moves the system even closer to the left instability in Fig 2B, thereby violating the temporal balance condition, Eq (6). This explains the occurrence of delta oscillations consistently observed in the stargazer model of absence epilepsy. This hypothesis can be tested directly by optogenetic interneuron and pyramidal neuron activation controlled by closed-loop, real-time recordings of pyramidal cell activity, a technique that is becoming well-established [24–27]. The prediction is that relative excess of slow (NMDA-like) optogenetically generated inward currents into interneurons over fast optogenetically generated (AMPA-like) currents into pyramidal neurons will result in delta oscillations and, when the imbalance is increased further, to seizure-like pathologies. Importantly, the model predicts that this occurs even though the balance of excitatory to inhibitory strengths is maintained.

### Model Dependence on NMDA Receptors

The dynamics observed in Figs 1 and 2 depend on the addition of NMDA receptors to the excitatory connections. A number of previous studies have considered response times in similar networks but without NMDA. They showed that in spiking networks response times on the order of or faster than the membrane time constant were possible and did not observe a delta oscillatory instability [4, 5]. In Fig 3 we show how the dynamics in Fig 2 depend upon the amount of NMDA on the excitatory projections, *q*, and the time constant of decay for the NMDA receptors, *τ*^*nmda*^. We consider a broad range of values for *τ*^*nmda*^ as has been previously observed [28–31] as well as values of *q* ranging from 0 as in previous computational work that did not consider NMDA receptors to NMDA/AMPA ratios that have been observed in cortex [31, 32].

Qualitative characteristics of the network dynamics are maintained for almost the entire range of both *q* and *τ*^*nmda*^. Fig 3A shows that the delta oscillatory instability is maintained for all parameter values except for a small region near *q* = 0. For example, when *τ*^*nmda*^ = 100 ms the network has no delta oscillatory instability for *q* ∈ [0, 0.01]. This is consistent with the stability observed in previous work but also shows that even small amounts of NMDA require consideration of the temporal balance condition. The oscillatory instability is also still in the delta range across the whole parameter regime, Fig 3B.

Fig 3C shows the rise time for each parameter set when Δ*q* is chosen such that the network is critically damped which we define as the point just before the poles involved in the delta oscillations separate from the real line. When the recurrent connections in the network have a higher percentage of AMPA receptors (smaller *q*) then the network generally responds more quickly to changes in input as would be expected from a network with faster synaptic responses. However, for some non-zero values of *q* the network actually responds more quickly than the network with no NMDA receptors, *i.e.* when *q* = 0. In these cases, each recurrent projection is slower then one with only AMPA receptors yet the negative value of Δ*q* at critical damping drives the system causing an overall reduction in rise time. Interestingly, this driving of the network for negative Δ*q* produces a counterintuitive interaction between rise time and *τ*^*nmda*^: longer decay times for the NMDA receptors produce a faster rise time in the critically damped network. In fact, the fastest rise time we observe in our simulations of linear networks, 52.5 ms, occurs when *q* = 0.004, Δ*q* = −0.003 and *τ*^*nmda*^ = 400 ms. Rise times on this order have been previously observed in visual cortex [33]. Faster rise time for all our networks are possible if shorter membrane time constants and faster *τ*^*ampa*^ and *τ*^*gaba*^ are used. Observation of an inverse relationship between rise time and *τ*^*nmda*^ in cortex would be an important experimental confirmation of our model.

Although fast responses are possible for a subset of values of Δ*q*, most of the stable values of Δ*q* sit in the overdamped, large rise time regime. This regime shows effectively linear increases in rise time as a function of Δ*q* as can be seen in the portion of the blue line in Fig 2D where Δ*q* > 0. In Fig 3D we show how the slope of this line depends upon *q* and *τ*^*nmda*^. Contrary to the critically damped network, in the overdamped network larger *τ*^*nmda*^ produces longer rise times for each positive increment in Δ*q* as would be predicted from slower synapses. However, the value of *q* has almost no impact. The fact that the value of *q* does not change the relationship between rise time and Δ*q* implies that even when *q* is zero the addition of NMDA receptors on EE connections can cause increases in the rise time of the network. Therefore, even in networks which start with no NMDA receptors, small additions of such receptors could still slow the response of the network significantly, making our analysis important even in such corner cases.

### STD and the Effective Network Time Constant

In the previous section we showed that the stability, rise time and oscillatory activity of a balanced network depend on the relative strengths of AMPA and NMDA receptors that form the EE and IE projections. Our analysis assumed that the synaptic strengths are static. However, most synapses experience changes in their effective strength through short term plasticity which, on excitatory projections, is dominated by STD [34, 35]. Since STD has a range of observed values, synapses with different AMPA/NMDA ratios may have STD with different strengths. Such a combined distribution of STD strength and AMPA/NMDA ratio will cause the effective value of *q* on each projection and therefore Δ*q* to change over time. We therefore study the balanced network model with non-uniform STD on excitatory projections, as described in Materials and Methods, Eqs (9)–(12).

We consider the evolution of the synaptic strength on excitatory projections due to STD by parameterizing synaptic dynamics around the base usage rate *u* = 0.2, defining *u*^*ampa*^ = *u* − Δ*u* and *u*^*nmda*^ = *u* + Δ*u*. We also use a recovery time constant of *τ*_*r*_ = 500 ms. The base STD parameters are taken from within distributions found in rat visual cortex (we are not aware of equivalent data in primates) [36]. The parameter Δ*u* models the same effect as Δ*q* but in a time dependent manner. As with Δ*q*, Δ*u* = 0 maintains the temporal balance condition while positive values of Δ*u* increase network damping and negative values yield an underdamped network. Fig 4A shows the behavior of the rate network for three different values of Δ*u*. The parameters of the network meet both the balance and the temporal balance conditions at *t* = 0, and as the network evolves it exhibits dynamics similar to the linear network (without STD) studied above. Negative Δ*u* causes the temporal balance condition to move towards the AMPA dominated unstable regime and the network begins to oscillate in the delta range. Positive Δ*u* yields an effective increase in the proportion of NMDA currents resulting in a greater network time constant, in which case the network moves further from the AMPA dominated instability.

Parameters of STD vary over a broad range in cortex [36]. Within the range of values of *u* and *τ*_*r*_ observed in that study, the qualitative dynamics of the network are the same as reported here, *viz* delta oscillations followed by instability for negative Δ*u*, and long time constant responses for positive Δ*u*, except for some deviations occurring for very strong STD. When *u* > 0.35 and *τ*_*r*_ > 1.5 s, the STD associated with a large initial spike dampens the subsequent response, thereby reducing the initial oscillatory activity seen in Fig 4A. However, the slow response for positive values of Δ*u* remains the same for strong STD. In addition, increasing values of *u* and *τ*_*r*_ require larger values of Δ*u* in order to produce similar dynamics due to weaker recurrent connections caused by the increasing strength of STD.

Since the strength of the connections between populations are constantly changing due to STD, the networks in Fig 4A have different response characteristics as a function of time. In order to visualize how STD impacts these networks we plotted the rise time and parameters of linear networks with the same instantaneous synaptic strengths as the nonlinear networks at each time point, Fig 4B and 4C. We define instantaneous synaptic strength as the values of *J* in Eqs (10)–(12). Fig 4B shows how the rise time of the linear networks change over time while Fig 4C shows how the decreasing synaptic strengths due to STD project onto two parameters of the linear network, the strength of the EE and the IE projections *W*_*e*_ (see figure caption) and Δ*q*. The regions of instability and the rise times plotted in Fig 4C are computed from the linear system using the synaptic strengths as defined in Materials and Methods. Although the network with STD is nonlinear, the approximate linear systems show qualitatively similar dynamics. When negative Δ*u* yields faster rise time the linear system at each time point also shows faster rise times, Fig 4B. The same is true for slow responses with positive values of Δ*u*. Additionally, as the linear networks cross into the delta oscillatory instability the nonlinear networks begin to oscillate as well, Fig 4A and 4C. This implies that the impact of STD on the network dynamics can be broadly understood using the temporal balance condition.

Results transfer to spiking neurons. Fig 4D shows how networks of LIF neurons respond to similar STD parameters as the rate network in Fig 4A. As in the network without STD, larger changes in the underlying parameters are required to produce similar dynamics.

We have shown that differences in the distribution of STD across EE and IE projections in a balanced network can produce significant changes in both network stability and response time (*τ*_*n*_). In addition to maintaining the base distributions of NMDA/AMPA receptors and synaptic strength, the network must have homeostatic mechanisms adjusting the joint distribution of parameters controlling STD and NMDA/AMPA ratios. Concurrent modulation of synaptic strength, AMPA/NMDA ratio and short term plasticity parameters has been shown to occur in cortical networks [37, 38]. Cortical networks have also been shown to carefully control the NMDA/AMPA ratio both at individual synapses and across cortical areas [31, 32]. In the next section we address the question of whether the necessary information is available to the network to implement such mechanisms.

### Delta Oscillations and Homeostasis

We have shown that many neocortical networks operate close to an oscillatory instability associated with the relative ratios of AMPA to NMDA currents in the EE and IE projections. The ratio is determined by distributions of synaptic variables across the population but individual neurons cannot have direct access to information about the distributions. However, since each neuron in a local network receives both a sampling of external inputs and of the recurrent output of the population, it has implicit access to the network state through the frequency response, defined as its output as a function of its input. As seen from an individual neuron, the network input is simply the external input, and the network output is the recurrent input to the neuron from the network. Given a large enough sample of these inputs and outputs, an individual neuron can thus obtain an approximation of the frequency response of the network. Since increasing delta oscillations indicate that the network is approaching instability, analysis of the frequency response allows corrective action to be taken to maintain network stability.

We tested whether individual neurons in a network of randomly connected LIF neurons can detect local network oscillations as Δ*q* is altered. We used a constant Poisson input to drive the network. An approximation of the frequency response was then obtained for each excitatory neuron using the discrete Fourier transform of its recurrent glutamatergic inputs, *i.e.* the network outputs (note that at this point, our interest is only in the availability of the information, not detailed biophysical mechanisms). The magnitude of the average frequency response across ten independent runs shows a clear peak in the delta range as the network approaches instability (Fig 5A). The mean area under the transfer function in the delta range [0.5Hz, 5.5Hz] shows a clear increase as Δ*q* becomes more negative (Fig 5C). A homeostatic mechanism based on this information could thus cause neurons to shift Δ*q* towards stability. Similar responses, although in a slightly higher frequency range, were observed when the instabilities were due to changing synaptic strengths caused by STD (Fig 5B and 5C), allowing regulation of Δ*u*.

## Discussion

Networks with balanced excitation and inhibition are ubiquitous in cortex and other brain areas. It is well-known that the relative strength of excitation and inhibition in these networks needs to be controlled to keep them stable and to maintain their functionality. We show that in addition to this constraint on overall synaptic strength, a second condition is required to maintain network stability. This “temporal balance condition” specifies the relative weight of (fast) AMPA and (slow) NMDA receptors in the balance of positive and negative feedback. Temporal balance provides a highly sensitive parameter for setting the response time of the network which can be adapted over a range from tens of milliseconds to seconds by small adjustments of the AMDA/NMDA ratio. The range of stability is bracketed by two different oscillatory singularities. Networks approaching the NMDA-dominated instability express gamma-range oscillations, of a type described previously. A novel finding is that AMPA-dominated networks close to instability oscillate in the delta range, a possible source of cortical delta waves and a potential cause of absence epilepsy. Finally, we show that individual neurons in the network have access to information that allows them to homeostatically tune the set point for temporal balance to the optimal range.

While previous work has shown that neurons in the asynchronous state could be driven to respond more quickly than their membrane time constant we show that different time constants on excitatory connections may also be able to speed up the network response [4, 5]. In Fig 3C the network responds more quickly to input when the excitatory connections have a small amount of long time constant NMDA receptors than if the excitatory connections only have AMPA receptors. As we note in the main text, this makes the counterintuitive prediction that longer NMDA decay times may cause cortical networks to respond more quickly.

Although a connection between fast network rise times and low frequency oscillations seems counterintuitive, recent work has made a connection between fast reaction times and delta oscillatory phase entrainment [45, 46]. The connection between delta oscillations and fast reaction times has been explained as increased excitability for neuronal populations when the phase of delta oscillations and stimulus onset are appropriately aligned [47]. Our work indicates that power in the delta range may also correlate with faster rise times in the underlying cortical networks. The connection between delta oscillations and rise time also implies an interesting trade-off. A network which responds most quickly to a sensory input may also have long periods of relative quiescence during the trough of each delta oscillation. Therefore, such a trade-off may only be useful when the timing of the sensory input is predictable [48]. In future work it would be interesting to examine the interaction between excitability, rise time and delta phase as a function of AMPA/NMDA ratios.

While the temporal balance condition is important in maintaining the stability of the underlying network it also allows for significant changes in the rise time. Previously it has been shown that, unlike positive feedback networks, the network in Fig 2A can change its gain without significantly altering its rise time [7]. Here we show that the converse is also true, such a network is able to alter its rise time without changing its gain, Fig 1C. Therefore, it may allow a network to change the temporal aspect of its neural code independently of its magnitude.

The model we use to examine the impact of STD on the temporal balance condition assumes that STD impacts AMPA and NMDA receptors differently. This could be viewed as having two populations of synapses with either AMPA or NMDA synapses and different values for STD. Although physiological evidence does not support such a binary model there is evidence to support a spatial distribution of each type of receptor across a neuron and a broad range of values for STD parameters [36, 50]. Additionally, LTP appears to induce transient changes in the AMPA/NMDA ratio which could be impacted by a distribution values of STD parameters [51]. How the AMPA/NMDA ratio is co- distributed with different STD parameters would determine the effect on the temporal balance condition. Future work on this subject should examine how different distributions of AMPA/NMDA ratios and STD parameters impact the rise time and stability of cortical networks.

Maintenance of such a network requires homeostatic mechanisms which ensure that both balance conditions are met. Much of the current work on homeostatic mechanisms in cortex has focused on global synaptic scaling which has been shown to maintain the balance between excitatory and inhibitory projections [52]. These global mechanisms are exactly what would be required for maintenance of the balance condition in derivative feedback networks. Some experimental paradigms have also shown that the strength of AMPA and NMDA currents scale proportionately or are co-regulated during scaling [32, 53, 54]. Such mechanisms may be able to act quickly to regulate individual synapses [55]. Other experimental work has shown that STD and the ratio of AMPA to NMDA receptors are concurrently regulated during LTP through both pre-synaptic and post-synaptic processes [56, 57]. These experiments show that at least in principle the mechanisms required for network level modulation of synaptic strength, STD and AMPA/NMDA ratio exist in neocortical synapses.

Although we use the frequency response to examine the stability of our network, a full Fourier transform is not necessary. Knowledge of the change in amplitude across a set of relevant frequencies is sufficient, Fig 5C. Such a homeostatic mechanism would only require appropriate bandpass filtering of the incoming signal. Many studies have shown that frequency selective calcium signalling is important in intracellular homeostasis and control [39–41]. Since, the oscillatory activity in our model is in part driven by calcium permeable NMDA receptors similar frequency selective mechanisms could be used to drive the homeostatic response. Additionally, frequency selectivity is a relatively general characteristic of chemical systems implying that other signalling pathways are also possible [42].

The scaling of AMPA/NMDA ratios implied by our mechanism is determined by the parameters of the network as a whole rather than of the individual neuron. Therefore, the homeostatic mechanism should operate on the synapses associated with independent subnetworks to which the neuron is connected rather then scaling across all synaptic connections for the neuron. For example, local recurrent connections within a cortical column could be one appropriate subnetwork. If that network shifts towards instability then only synapses connected to other neurons in that network should be impacted. If an individual neuron is connected to multiple networks then the stability of each network should be approached separately. Although an examination of homeostatic mechanisms as a function of network connectivity is not available, synapse specific homeostatic scaling has been observed in previous work [43]. Additionally, target-specific short-term plasticity has been shown to exist in cortex implying that information about network level connectivity may be available to individual neurons [44].

It is generally assumed that synaptic strengths form an essential part of long-term memory. It therefore is natural to ask how the homeostatic mechanisms described here interact with the potential storage of memory contents. If, as is usually assumed, the information contained in each synapse is primarily related to its steady-state strength, then our mechanism should have minimal impact on memory contents and coding efficacy. The proposed frequency-based homeostatic mechanism while altering the AMPA/NMDA ratio does not alter the steady state value of each synapse for a given input. It only changes the transient activity of the synapse on the time scale of the NMDA receptors. Therefore, learning rules, read-out mechanisms *etc.*, as long as they are defined in terms of steady-state synaptic strengths, will be unaffected by the homeostatic adjustments.

In this work, our model of NMDA does not include voltage dependence. A rate based approximation to voltage dependence has been developed and would provide a more physiologically plausible model [49]. However, when there are different amounts of NMDA on the EE and IE connections, the rate dependence of NMDA receptors tends to have a greater impact on the balance between excitation and inhibition than the relative timing of the feedback. Although maintaining such a balance must be an ubiquitous requirement in cortex and is not unique to our model, studying the excitatory/inhibitory balance condition was not the focus of our work. The implications of this additional nonlinearity would be an interesting topic for a future study.

## Materials and Methods

### Reduced Rate-Based Network

The reduced rate-based network depicted in Fig 1A is defined by five ordinary differential equations, a simplified version of the full network defined by Eqs (2)–(4):

$$\begin{array}{ll} \tau_{e}\frac{dR}{dt}=-R & +w\left(\left(1-q-\Delta q\right)S_{+}^{ampa}+\left(q+\Delta q\right)S_{+}^{nmda}\right)\\ & -w\left(\left(1-q\right)S_{-}^{ampa}+qS_{-}^{nmda}\right)+I\left(t\right) \end{array}$$ (7)

$$\tau_{m}^{l}\frac{dS_{m}^{l}}{dt}=-S_{m}^{l}+R$$ (8)

where *R* represents the firing rate of the population, with intrinsic time constants *τ*_*e*_. There are two recurrent projections, one excitatory and one inhibitory, each with a total synaptic weight *w*. $S_{m}^{l}$ represents the synaptic activation of these two projections. *m* is the projection type, + for excitatory and − for inhibitory, and *l* is the synapse type, either AMPA or NMDA. In the diagram in Fig 1A, fast and slow are respectively equivalent to AMPA and NMDA in this formulation of the model. The parameter *q* is the proportion of each projection that is carried by the NMDA receptors. Δ*q* represents a change in the ratio of AMPA and NMDA receptors on the excitatory projection relative to the inhibitory projection. Time varying input to the neuronal population is denoted by *I*(*t*). In the simulations in Fig 1C, middle row, we use *w* = 30, *q* = 0.3, *τ*_*e*_ = 20 ms, *τ*^*ampa*^ = 5 ms and *τ*^*nmda*^ = 100 ms. For the plots from left to right Δ*q* is −0.0425, −0.0340, −0.0095 and 0.125. The value of Δ*q* for the undamped oscillator was computed analytically from the characteristic polynomial, see Supplement. Parameters for critical damping were found by looking for the emergence of two complex roots near the point of instability. The values for the underdamped and overdamped systems were chosen to make the dynamics similar to the simulations from the spring approximation.

### Full Rate-Based Network

The dynamics of the network are described by eight ordinary differential equations, Eqs (2)–(4). We consider time varying input to the excitatory population *I*(*t*) given by a step input of amplitude 5 Hz for the linear network and 10 Hz for the network with STD filtered by a 100ms Gaussian kernel. The time constants are: *τ*_*e*_ = 20 ms, *τ*_*i*_ = 10 ms, *τ*^*ampa*^ = 5 ms, *τ*^*nmda*^ = 100 ms and *τ*^*gaba*^ = 10 ms. The values of the firing rate variables, *R*_*e*_ and *R*_*i*_, are constrained to always be positive except in Fig 1. The synaptic strengths are redefined in terms of *w*, *k*, *q* and Δ*q* (see section on Rise Time and Stability in the Linear Network) to reduce the dimensionality of the parameter space and to automatically enforce the balance conditions. For all networks without STD we set *q* = 0.3. In the simulations in Fig 1C bottom row we use *w* = 30, *k* = 1.5 and from left to right Δ*q* is −0.0226, −0.0196, −0.0068 and 0.05. The value of Δ*q* for the undamped oscillator was found by searching for a root with zero real part as a function of Δ*q*. Values for critical, overdamped and underdamped systems were found as for the reduced networks, see section on the Reduced Rate-Based Network.

### Networks with Short Term Depression

We use the STD mechanism described in ref. [58] for excitatory synapses (there is no STD on inhibitory projections):

$$\frac{dx^{l}}{dt}=\frac{1-x^{l}}{\tau_{r}^{l}}-u^{l}x^{l}R\left(t\right)$$ (9)

where *x*^*l*^(*t*) ∈ [0, 1] represents the depressed synaptic efficacy due to STD, $\tau_{r}^{l}$ is the recovery rate of the synapse. The instantaneous rate of the input to the synapse is *R*(*t*) and *u*^*l*^ is the usage rate which is a constant in our formulation. *l* is either AMPA or NMDA. Therefore, we have one Eq (9) for *x*^*l*^ = *x*^*ampa*^ and another for *x*^*l*^ = *x*^*nmda*^. We redefine the synaptic strengths of excitatory connections in Eqs (2) and (3) to include STD,

$$J_{ee}^{ampa}=\frac{1}{2}x^{ampa}w$$ (10)

$$J_{ee}^{nmda}=\frac{1}{2}x^{nmda}w$$ (11)

$$J_{ie}^{ampa}=J_{ie}^{nmda}=\frac{\left(x^{ampa}+x^{nmda}\right)}{4}w$$ (12)

Evolution of *x*^*ampa*^ and *x*^*nmda*^ is determined by Eq (9) with parameters *u*^*ampa*^, $\tau_{r}^{ampa}$, *u*^*nmda*^ and $\tau_{r}^{nmda}$. We define Δ*u* = (*u*^*nmda*^ − *u*^*ampa*^)/2. Since we are predominantly interested in the effect of STD on the temporal balance condition, we set STD on the IE projections to exactly balance the EE projections without changing the effective time constant. The balance condition is always met if *q* = 0.5 and STD on the IE projections is the average of *x*^*ampa*^ and *x*^*nmda*^, Eqs (10)–(12). This also ensures that *q* remains constant and changes in the parameters of *x*^*ampa*^ and *x*^*nmda*^ only change Δ*q*.

### LIF Networks

All our LIF networks have *N*_*e*_ = 3,200 excitatory and *N*_*i*_ = 800 inhibitory neurons. Each neuron is represented by the standard LIF equation and synapses have exponentially decaying activation,

$$\tau \frac{dV_{m}}{dt}=-\left(V_{m}-E_{l}\right)+\sum_{n,l}J_{mn}^{l}S_{mn}^{l}+I\left(t\right)+\eta \left(t\right)$$ (13)

$$\tau_{mn}^{l}\frac{dS_{mn}^{l}}{dt}=-S_{mn}^{l}+\sum_{n,k}\delta \left(t-t_{mn}^{\alpha }\right)$$ (14)

where *τ* is the membrane time constant and takes the values *τ*_*e*_ for excitatory and *τ*_*i*_ for inhibitory neurons as before. *E*_*l*_ = −60 mV is the reversal potential, *V*_*m*_ is the membrane voltage for neuron *m* with firing threshold −40 mV and a reset potential after firing of −52 mV. $J_{mn}^{l}$ is the strength of the synapse from neuron *n* to neuron *m* on receptor type *l*, taking the value *w*/(*N*_*e*_*p*) for excitatory synapses and *w*/(*N*_*i*_*p*) for inhibitory synapses. The probability of making a connection from any neuron to any other is *p* = 0.2 and the proportion of AMPA and NMDA receptors is redefined by *q* and Δ*q* as in the rate model. $S_{mn}^{l}$ is the synaptic activation, $t_{mn}^{\alpha }$ is the time of the *α*-th incoming action potential at synapse *mn* and $\tau_{mn}^{l}$ is the decay time for type *l* of synapse where *τ*^*gaba*^ = 10 ms and *τ*^*nmda*^ = 100 ms as before. *τ*^*ampa*^ was increased to 10 ms to improve the stability of the network. For the simulations in Fig 2, each neuron receives Poisson-distributed background input *η*(*t*) from 1,000 neurons with event strength 0.2 mV. The background input to excitatory neurons has firing rate 1.05 Hz and to inhibitory neurons 1.0 Hz; these values were chosen to maintain the same baseline firing rate for both populations. In addition, at *t* = 0 s the voltage of the excitatory population receives a step input of *I*(*t*) = 3 mV filtered by a 100 ms Gaussian kernel. All spiking simulations were run using the Brian spiking neural network simulator [59].

For LIF neurons with STD, *R*(*t*) in Eq (9) is replaced by $\sum_{n,k}\delta \left(t-t_{mn}^{k}\right)$. The *p* = 0.2 connection probability is evenly split between an AMPA and an NMDA projection, effectively giving *q* = 0.5. The usage rates, *u*^*ampa*^ and *u*^*nmda*^ on the EE projections differ by Δ*u* as in the rate model. On the IE projections, the sign of Δ*u* is reversed such that, Δ*u*_*ie*_ = −Δ*u*. For the simulations in Fig 4, neurons receive the same background stimulation as for the LIF network without STD. At *t* = 0 the voltage of the excitatory population receives a step input of *I*(*t*) = 6 mV filtered by a 100 ms Gaussian kernel ending at *t* = 1 s.

The frequency response in the LIF network with and without STD is computed from a 4 second window of spiking activity in both networks. Each neuron receives the same background activity as in previous simulations. The network without STD receives a 2 mV step input and runs for 4 seconds prior to the window. The network with STD receives a 12 mV step input and runs for 1.5 seconds prior to the window.

## Supporting Information

S1 FigStability, rise time and oscillatory activity of the rate based model as a function of the network parameters.All networks use *q* = 0.30 unless otherwise noted. The white crosses on A-F represent the values of *k* and *w* used for the rate based network without STD in the main text. A: Change in *q* on the EE projection required to reach the AMPA dominated instability. The colorbar refers to negative values of Δ*q*. B: Change in *q* on the EE projection required to reach the bifurcation yielding delta oscillations. The colorbar refers to negative values of Δ*q*. C: The rise time in seconds at which the network begins to produce delta oscillations. D: Change in *q* on the EE projection required to reach the NMDA dominated instability. E: Rise time of the network for a constant value of Δ*q*. Shows the slope of the rise time as a function of *k* and *w*. Δ*q* = 0.075 was chosen to ensure that all instantiations of the network were stable and had minimal oscillations. F: Location of the peak in the frequency response as the network approaches the AMPA dominated instability. G: Location of the peak in the frequency response as the network approaches the AMPA dominated instability. Network parameters were *k* = 1.2 and *w* = 30. *q* is the proportion of synaptic strength through NMDA receptors. *f*^*τ*^ is a reduction in the membrane time constant of the excitatory and inhibitory neurons such that $\tau_{e}^{new}=f^{\tau }\tau_{e}$ and $\tau_{i}^{new}=f^{\tau }\tau_{i}$. H: Poles of the rate based network without STD plotted as a function of Δ*q*. The imaginary axis is in units of Hz. The right panel is an expansion of the box in the left panel (black rectangle around the origin). Poles cross the imaginary axis for large positive Δ*q* at about 60 Hz, corresponding to an oscillatory instability in the gamma range (blue circles, left panel), and for small negative Δ*q* at about 2 Hz, corresponding to an oscillatory instability in the delta range (red circles, right panel).(TIF)

S1 Text Contains all of the supplementary modelling associated with the main text including a discussion of the supplementary figure. (PDF)
