# Supplementary figures and images for: The Edge of Stability: Response Times and Delta Oscillations in Balanced Networks
Source: PLoS Comput Biol. 2016 Sep 30;12(9):e1005121. doi: 10.1371/journal.pcbi.1005121 (PMC5045166; doi:10.1371/journal.pcbi.1005121)

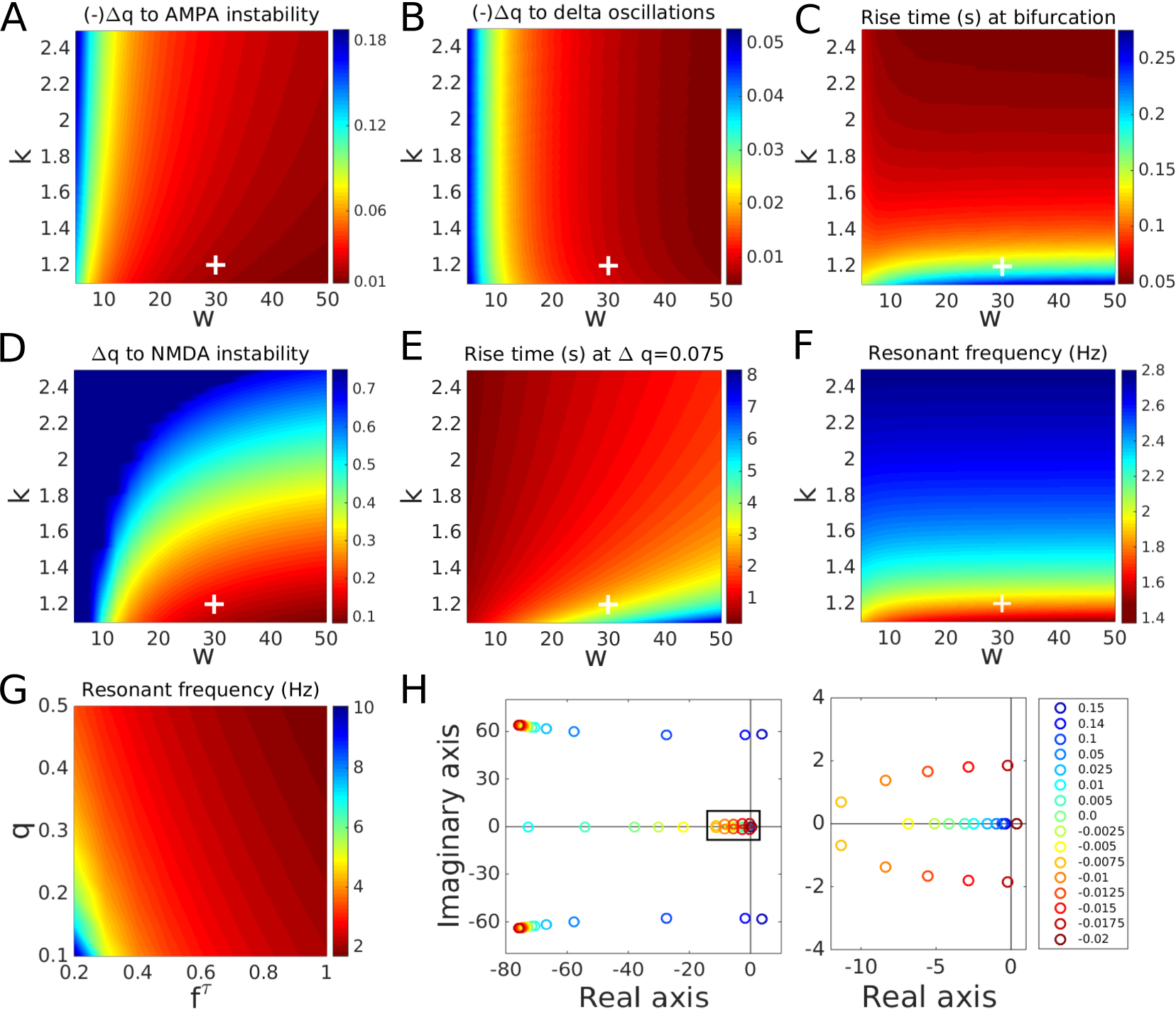

Supplement: S1 Fig — All networks use q = 0.30 unless otherwise noted. The white crosses on A-F represent the values of k and w used for the rate based network without STD in the main text. A: Change in q on the EE projection required to reach the AMPA dominated instability. The colorbar refers to negative values of Δq. B: Change in q on the EE projection required to reach the bifurcation yielding delta oscillations. The colorbar refers to negative values of Δq. C: The rise time in seconds at which the network begins to produce delta oscillations. D: Change in q on the EE projection required to reach the NMDA dominated instability. E: Rise time of the network for a constant value of Δq. Shows the slope of the rise time as a function of k and w. Δq = 0.075 was chosen to ensure that all instantiations of the network were stable and had minimal oscillations. F: Location of the peak in the frequency response as the network approaches the AMPA dominated instability. G: Location of the peak in the frequency response as the network approaches the AMPA dominated instability. Network parameters were k = 1.2 and w = 30. q is the proportion of synaptic strength through NMDA receptors. fτ is a reduction in the membrane time constant of the excitatory and inhibitory neurons such that τenew=fττe and τinew=fττi. H: Poles of the rate based network without STD plotted as a function of Δq. The imaginary axis is in units of Hz. The right panel is an expansion of the box in the left panel (black rectangle around the origin). Poles cross the imaginary axis for large positive Δq at about 60 Hz, corresponding to an oscillatory instability in the gamma range (blue circles, left panel), and for small negative Δq at about 2 Hz, corresponding to an oscillatory instability in the delta range (red circles, right panel). (TIF) [file pcbi.1005121.s001.tif]
