# Supplementary material for: The Edge of Stability: Response Times and Delta Oscillations in Balanced Networks
Source: PLoS Comput Biol. 2016 Sep 30;12(9):e1005121. doi: 10.1371/journal.pcbi.1005121 (PMC5045166; doi:10.1371/journal.pcbi.1005121)
Supplement: S1 Text — (PDF) [file pcbi.1005121.s002.pdf]

Supplementary Modelling

The Edge of Stability:
Response Times And Delta Oscillations in Balanced Networks

Grant Gillary [1,2], Ernst Niebur [1,2*],

1 Zanvyl Krieger Mind/Brain Institute, Baltimore, MD, USA.
2 Solomon Snyder Department of Neuroscience, Johns Hopkins University, Baltimore, MD, USA

* niebur@jhu.edu

1 Stability in the Single Population Model

The single population model is depicted in **Fig 1A** and its equations formulated in the Materials and Methods section, Eqs (7)-(8). This model maintains all of the dynamics of interest from the full balanced network as a function of Δq including the delta oscillatory instability, fast response to step input and the subsequent slowing of the network response for large positive values of Δq. This implies that the integration of the inhibitory population and the disinhibitory feedback are not necessary to produce network behavior of this type. However, the removal of the inhibitory population and its disinhibitory feedback does reduce the overall dynamical range of the network. The single population model no longer produces balanced amplification nor does it have a gamma oscillatory instability when Δq is very large and positive. However, both of these types of dynamics have been described previously [1, 2] and are not the focus of our work.

The single population model can be reduced to a third order system by recognizing that each of the four synaptic equations has the same input, $R(t)$. Therefore, setting $S_+^{ampa} = S_-^{ampa}$ and $S_+^{nmda} = S_-^{nmda}$ reduces the system to third order. After canceling out terms the new single population model is:

$$\tau_e \frac{dR}{dt} = - R + w\Delta q \left(S^{nmda} - S^{ampa}\right) + I(t) \tag{S1}$$

$$\tau^l \frac{dS^l}{dt} = - S^l + R \tag{S2}$$

where R represents the firing rate of the population, with intrinsic time constant τ_e, and w is the synaptic weight. S^l represents the synaptic activation which decays exponentially with time constant τ^l. l is the synapse type, either AMPA or NMDA. Δq represents a change in the ratio of AMPA and NMDA receptors on the excitatory projection relative to the inhibitory projection. All information about the excitatory and inhibitory projections is now combined into the $w\Delta q$ term indicating that most of the recurrent excitation and inhibition has canceled with only the difference in timing remaining. We can compute the transfer function of this system by taking the Laplace

transform of each equation and then combining. The transfer function of the single population system is:

$$R(\gamma) = \frac{1}{\tau_e} \frac{\left(\gamma + \frac{1}{\tau^{nmda}}\right)\left(\gamma + \frac{1}{\tau^{ampa}}\right)}{\gamma^3 + \left(\frac{1}{\tau^{nmda}} + \frac{1}{\tau^{ampa}} + \frac{1}{\tau_e}\right)\gamma^2 + \left(\frac{1}{\tau^{nmda}\tau^{ampa}} + \frac{1-w\Delta q}{\tau^{nmda}\tau_e} + \frac{1+w\Delta q}{\tau^{ampa}\tau_e}\right)\gamma + \frac{1}{\tau^{nmda}\tau^{ampa}\tau_e}} \tag{S3}$$

Instabilities of the network can be found by computing the roots of the denominator in Eq (S3). Any root with positive real part is unstable while a root with a real part of zero is marginally stable. Rather than look for all of the roots of the polynomial we search for a set of parameters which make the system marginally stable by assuming that $\gamma = \omega_0 j$ where j is imaginary and ω_0 is the oscillatory frequency in radians per second. Inserting $\omega_0 j$ into the polynomial gives,

$$\frac{1}{\tau^{nmda}\tau^{ampa}\tau_e} - \left(\frac{1}{\tau^{nmda}} + \frac{1}{\tau^{ampa}} + \frac{1}{\tau_e}\right)\omega_0^2 + \left(\frac{1}{\tau^{nmda}\tau^{ampa}} + \frac{1-w\Delta q}{\tau^{nmda}\tau_e} + \frac{1+w\Delta q}{\tau^{ampa}\tau_e} - \omega_0^2\right)\omega_0 j = 0. \tag{S4}$$

We then solve for the values of $w\Delta q$ and ω_0 which set the real part of the equation to zero. This gives the oscillatory frequency of the single population model at instability,

$$\omega_0 = \frac{1}{\sqrt{\tau^{nmda}\tau^{ampa} + \tau^{nmda}\tau_e + \tau^{ampa}\tau_e}}, \tag{S5}$$

as well as the value of $w\Delta q$ at instability,

$$w\Delta q = \frac{\tau^{nmda}\tau^{ampa}\tau_e}{\left(\tau^{nmda} - \tau^{ampa}\right)\left(\tau^{nmda}\tau^{ampa} + \tau^{nmda}\tau_e + \tau^{ampa}\tau_e\right)} - \frac{\tau^{nmda} + \tau^{ampa} + \tau_e}{\tau^{nmda} - \tau^{ampa}} \tag{S6}$$

The value of $w\Delta q$ given by Eq (S6) is used for the simulation of undamped oscillations in the main text, **Fig 1C**, left column, middle row.

2 Derivation of the Spring Approximation

In the main paper we compare the dynamical response of the full balanced network to a damped oscillator. This approximation is derived directly from the single population model which we described in the previous section. We begin by rewriting Eq (S3) . Multiplying the top and bottom of Eq (S3) by $\tau_e\tau^{nmda}\tau^{ampa}$ then dividing the top and bottom by $(\tau^{nmda}\gamma + 1)(\tau^{ampa}\gamma + 1)$ yields,

$$R(\gamma) = \frac{1}{\tau_e\gamma + 1 + \frac{w\Delta q(\tau^{nmda} - \tau^{ampa})\gamma}{(\tau^{nmda}\gamma+1)(\tau^{ampa}\gamma+1)}}. \tag{S7}$$

We then assume that the AMPA connections act instantaneously and therefore take the limit as τ^{ampa} approaches zero then rearrange into the standard form,

$$\lim_{\tau^{ampa}\to 0} R(\gamma) = \frac{1}{\tau_e\gamma + 1 + \frac{w\Delta q \tau^{nmda}\gamma}{\tau^{nmda}\gamma+1}} \tag{S8}$$

$$= \frac{1}{\tau_e}\frac{\gamma + \frac{1}{\tau^{nmda}}}{\gamma^2 + \frac{\tau_e+(1+w\Delta q)\tau^{nmda}}{\tau^{nmda}\tau_e}\gamma + \frac{1}{\tau^{nmda}\tau_e}}. \tag{S9}$$

The denominator of this transfer function describes a damped oscillator with dynamics given by the differential equation

$$\frac{d^2R(t)}{dt^2} + 2\zeta\omega_0\frac{dR(t)}{dt} + \omega_0^2 R(t) = I(t). \tag{S10}$$

where ω_0 and ζ are defined as,

$$\omega_0 = \frac{1}{\sqrt{\tau^{nmda}\tau_e}}, \tag{S11}$$

$$\zeta = \frac{1}{2}\frac{\tau_e + (1 + w\Delta q)\,\tau^{nmda}}{\sqrt{\tau^{nmda}\tau_e}}, \tag{S12}$$

and $I(t)$ is some time dependent input. Note that although we have taken the limit as τ^{ampa} goes to zero this is not equivalent to removing the AMPA portion of the model thereby only having one time constant on each projection. Instead the new network is one where the impact of the AMPA receptors are instantaneous and is still effectively represented by the network shown in **Fig 1A** in the main text.

As can be seen from Eq (S12), the damping coefficient ζ is a linear function of $w\Delta q$. This implies that Δq can be used as a reasonable approximation to ζ and that its impact on the network should be roughly equivalent. Given this damped oscillator formulation we can also compute the value of $w\Delta q$ where the network is critically damped by setting $\zeta = 1$ yielding $w\Delta q = (2\sqrt{\tau^{nmda}\tau_e} - \tau^{nmda} - \tau_e)/\tau^{nmda}$. Additionally, we can determine the point at which the system becomes marginally stable by setting $\zeta = 0$. We also call this the undamped network in the main text. The spring network is marginally stable when $w\Delta q = -(1 + \tau_e/\tau^{nmda})$. These equations for the critically damped and undamped networks were used to compute the values of $w\Delta q$ in **Fig 1C**, top row, used in the simulations. These equations also give an intuition for why the temporal balance condition as described in the main text does not exactly determine the stability of the network. For Eq (S12) to become less than zero, the $w\Delta q\tau^{nmda}$ term in the numerator of that equation must be negative and have its absolute value greater than the $\tau_e + \tau^{nmda}$ portion of the numerator. Both τ_e and τ^{nmda} represent damping caused by both the cell bodies of the neurons and the passing of the recurrent activity through the synapses. For the network to become unstable the transient imbalances caused by the $w\Delta q\tau^{nmda}$ term must drive the system sufficiently to counteract the inherent damping in the cell body and synapses.

3 Steady States of the Full Linear Network

The full linear network is defined in Eqs (2)-(4) in the main text. The steady states of the network can be found by setting the derivatives to zero and then solving for R_e, R_i

and S^l_{mn}. The steady state rates are,

$$
\begin{aligned}
R_e &= \frac{I(1+J_{ii})}{J_{ei}(J^{ampa}_{ie}+J^{nmda}_{ie})-(1+J_{ii})(J^{ampa}_{ee}+J^{nmda}_{ee}-1)},\\
R_i &= \frac{I(J^{ampa}_{ie}+J^{nmda}_{ie})}{J_{ei}(J^{ampa}_{ie}+J^{nmda}_{ie})-(1+J_{ii})(J^{ampa}_{ee}+J^{nmda}_{ee}-1)},\\
S^l_{mn} &= R_n.
\end{aligned} \tag{S13}
$$

As long as the balance conditions are met, the steady state equations for this network have only one solution and if the input I is zero the steady state is also zero. These equations do not depend upon any time constants or the NMDA/AMPA ratio implying that Δq does not impact the steady state firing rates. Since the network is linear it can be fully described by its coefficient matrix. The eigenvectors and eigenvalues of this matrix are the solution of the homogeneous system of linear ordinary differential equations. Since the eigenvalues represent the time constants of the network we will next consider how the eigenvalues of the coefficient matrix impact τ_n, the effective network time constant.

4 Network Time Constants

The roots of the characteristic polynomial of the balanced network

$$s^n + a_{n-1}s^{n-1} + \ldots + a_1 s + a_0 = 0. \tag{S14}$$

yield the eigenvalues. Using the coefficients of Eq (S14) it can be shown that the first eigenvalue is small and negative when the ratio a_1/a_0 is large and positive, yielding a large network time constant τ_n [3]. Conversely, when a_1/a_0 is small and positive then the first eigenvalue will be large and negative, resulting in a short τ_n. We do not provide sufficient conditions for small τ_n, only a necessary condition. However, simulations show that when a_1/a_0 is small and positive, τ_n is also small, **Fig 2D**. We assume that J is large with respect to the leak current and that all synaptic strengths, J_{mn}, are of order J. Due to the large number of terms in the characteristic equation we only consider coefficients in the highest order of J. Using Mathematica (Wolfram Inc, Champaign IL) we calculate the coefficients of the characteristic equation in highest order (J^2), yielding,

$$a_0 = J_{ei}(J^{ampa}_{ie}+J^{nmda}_{ie}) - J_{ii}(J^{ampa}_{ee}+J^{nmda}_{ee}), \tag{S15}$$

$$
\begin{aligned}
a_1 = &J_{ei}(J^{ampa}_{ie}+J^{nmda}_{ie})(\tau^{ampa}_{ee}+\tau^{nmda}_{ee}+\tau^{gaba}_{ii})\\
&- J_{ii}(J^{ampa}_{ee}+J^{nmda}_{ee})(\tau^{ampa}_{ie}+\tau^{nmda}_{ie}+\tau^{gaba}_{ei})\\
&+ J_{ei}(J^{ampa}_{ie}\tau^{nmda}_{ie}+J^{nmda}_{ie}\tau^{ampa}_{ie})\\
&- J_{ii}(J^{ampa}_{ee}\tau^{nmda}_{ee}+J^{nmda}_{ee}\tau^{ampa}_{ee}).
\end{aligned} \tag{S16}
$$

The ratio a_1/a_0 is large, thereby producing a large τ_n, if the following three conditions are met,

$$J_{ei}(J^{ampa}_{ie}+J^{nmda}_{ie}) - J_{ii}(J^{ampa}_{ee}+J^{nmda}_{ee}) \ll O(J^2), \tag{S17}$$

$$J_{ei}(J^{ampa}_{ie}+J^{nmda}_{ie})(\tau^{ampa}_{ee}+\tau^{nmda}_{ee}+\tau^{gaba}_{ii}) -$$

$$J_{ii}(J_{ee}^{ampa} + J_{ee}^{nmda})(\tau_{ie}^{ampa} + \tau_{ie}^{nmda} + \tau_{ei}^{gaba}) \ll O(J^2), \quad \text{(S18)}$$

$$J_{ei}(J_{ie}^{ampa}\tau_{ie}^{nmda} + J_{ie}^{nmda}\tau_{ie}^{ampa}) - \\ J_{ii}(J_{ee}^{ampa}\tau_{ee}^{nmda} + J_{ee}^{nmda}\tau_{ee}^{ampa}) \sim O(J^2). \quad \text{(S19)}$$

These three conditions are taken directly from terms in Eqs (S15) and (S16) using the requirement that a_1/a_0 is large. The first constraint, Eq (S17), states that positive feedback (second term) and negative feedback (first term) should be balanced. This "balance condition" is also a requirement for the system to be stable as we will see in a later section. The other two constraints describe the "temporal balance condition," first introduced in this contribution. Formula (S18) describes a condition which depends on the overall time constants of the EE and IE connections. It produces changes in the temporal balance condition if receptors on each projection have different time constants, for example, if $\tau_{ee}^{nmda} \neq \tau_{ie}^{nmda}$. However, if the sum of the time constants of the EE projection is the same as the sum of the time constants on the IE projection, then inequality (S18) reduces to the balance condition, inequality (S17), with a constant coefficient. This term has been described previously for networks with only one time constant on each projection [3]. We assume that synapses of the same neurotransmitter type have the same time constant on all projections, so τ^{ampa} is the same for all projections with AMPA currents, and the same applies to τ^{nmda} and τ^{gaba}. Therefore, Eq (S18) reduces to Eq (S17) and has little impact on τ_n, leaving only Eq (S19).

Eq (S19) describes the impact of changing the ratio of two time constants on the EE and IE projections which is how our network produces large τ_n as Δq increases. Fundamentally, this constraint states that changing the strength of AMPA and NMDA currents on the EE or IE projections will change the average time constant of that projection and thereby alter τ_n. In a mathematical sense, the cross multiplication of synaptic time constants for one type of synapse with the synaptic strength of the other type of synapse means that the first term in Eq (S19) can be made larger than the second term without breaking the balance condition. For example, if we increase J_{ee}^{nmda} by ΔJ and decrease J_{ee}^{ampa} by ΔJ then the balance condition is maintained. Additionally, $\Delta J\tau_{ee}^{ampa} < \Delta J\tau_{ee}^{nmda}$ which implies that the second term in Eq (S19) is decreased by the changes in synaptic strength. Another way to state this is that if $\tau_{ee}^{nmda} > \tau_{ee}^{ampa}$ then,

$$(J_{ee}^{ampa} - \Delta J)\tau_{ee}^{nmda} + (J_{ee}^{nmda} + \Delta J)\tau_{ie}^{ampa} < \\ J_{ee}^{ampa}\tau_{ee}^{nmda} + J_{ee}^{nmda}\tau_{ee}^{ampa}. \quad \text{(S20)}$$

This change increases the ratio a_1/a_0 yielding a longer time constant of decay. In addition, as can be seen from Eq (S13), this type of balanced change in synaptic strength will not change the equilibrium state of the network.

5 Stability

In addition to finding the eigenvalues of the system, the characteristic polynomial can also be used to determine the stability of the system. The Routh-Hurwitz stability criterion provides necessary and sufficient conditions for the stability of a time invariant linear system with constant coefficients. However, due to the complexity of the inequalities this yields we do not require that the polynomials be Hurwitz, but only that the coefficients of the characteristic polynomial are all positive which is a necessary but not sufficient condition for stability. The stability conditions below work well for the delta oscillatory instability when Δq is small but miss the gamma oscillatory instability for large and positive Δq.

The stability criteria are,

$$J_{ei}(J_{ie}^{ampa} + J_{ie}^{nmda}) > J_{ii}(J_{ee}^{ampa} + J_{ee}^{nmda}) \tag{S21}$$

$$\frac{J_{ii}}{\tau_i \tau_{ii}} > \frac{J_{ee}^{ampa}}{\tau_e \tau_{ee}^{ampa}} + \frac{J_{ee}^{nmda}}{\tau_e \tau_{ee}^{nmda}} \tag{S22}$$

$$\frac{J_{ii}}{\tau_i \tau_{ii}} \sum_{m \neq i, ii} \frac{1}{\tau_m^l} > \frac{J_{ee}^{ampa}}{\tau_e \tau_{ee}^{ampa}} \sum_{\substack{m \neq e, \\ (m,l) \neq \\ (ee,ampa)}} \frac{1}{\tau_m^l} + \frac{J_{ee}^{nmda}}{\tau_e \tau_{ee}^{nmda}} \sum_{\substack{m \neq e, \\ (m,l) \neq \\ (ee,nmda)}} \frac{1}{\tau_m^l} \tag{S23}$$

$$J_{ei}\left(J_{ie}^{nmda}\tau_{ie}^{ampa} + J_{ie}^{ampa}\tau_{ie}^{nmda}\right) > J_{ii}\left(J_{ee}^{nmda}\tau_{ee}^{ampa} + J_{ee}^{ampa}\tau_{ee}^{nmda}\right), \tag{S24}$$

$$\tau_{ii} + \tau_{ee}^{ampa} + \tau_{ee}^{nmda} > \tau_{ei} + \tau_{ie}^{ampa} + \tau_{ie}^{nmda} \tag{S25}$$

$$\tau_{ii}\, \tau_{ee}^{ampa}\tau_{ee}^{nmda} > \tau_{ei}\, \tau_{ie}^{ampa}\tau_{ie}^{nmda} \tag{S26}$$

$$\tau_{ii}(\tau_{ee}^{ampa} + \tau_{ee}^{nmda}) + \tau_{ee}^{ampa}\tau_{ee}^{nmda} > \tau_{ei}(\tau_{ie}^{ampa} + \tau_{ie}^{nmda}) + \tau_{ie}^{ampa}\tau_{ie}^{nmda}. \tag{S27}$$

The first condition, eq. (S21), requires that negative feedback be greater than positive feedback; this is the balance condition. We use it and the assumption that the time constant of particular receptor subtypes does not change in the derivation of Eqs (S25), (S26) and (S27). The next two conditions, Eqs (S22) and (S23), require that the time constant weighted strength of disinhibition be greater than that of pure positive feedback. In addition, the right side of both of these inequalities will be dominated by the AMPA synapses due to their small time constant. The fourth constraint, Eq (S24), is the temporal balance condition. The last three constraints show that the global positive feedback must have a longer time constant than the negative feedback. Additionally, the combination of Eqs (S25) and (S26) puts limits on the range of the time constants in the EE connections versus the IE connections.

The stability equations also constrain the possible time constants on each projection. One very strict example can be seen when we assume that $J_{ie}^{ampa} = J_{ie}^{nmda} = J_{ee}^{ampa} = J_{ee}^{nmda}$ and that $J_{ei} = J_{ii}$. This turns Eq (S24) into a new stability condition,

$$\tau_{ie}^{ampa} + \tau_{ie}^{nmda} > \tau_{ee}^{ampa} + \tau_{ee}^{nmda}. \tag{S28}$$

Given that $\tau_{ii} = \tau_{ei}$, this directly contradicts Eq (S25) and there is only a small subset of average synaptic time constants that are allowed. These time constants must meet two conditions,

$$\tau_{ie}^{ampa} + \tau_{ie}^{nmda} = \tau_{ee}^{ampa} + \tau_{ee}^{nmda}, \tag{S29}$$

$$\tau_{ee}^{ampa}\tau_{ee}^{nmda} > \tau_{ie}^{ampa}\tau_{ie}^{nmda}. \tag{S30}$$

The strict inequality in Eq (S25) implies that such a system should be unstable. However, in practice equality in the stability conditions also gives a stable network which is likely due to the fact that these constraints themselves are approximations. In general the numerically computed roots from the coefficient matrix show a larger area of stability than the heuristics we have provided. What Eqs (S25)-(S27) seem to imply

is that changing the ratio of synaptic strengths of the different receptors on the excitatory projections is a more stable way to alter the effective time constant of a projection than changing the time constants of receptors.

6 Coefficients of the Characteristic Polynomial

In the previous section we examined the stability conditions associated with the rate based balanced network. This analysis depends upon two types of approximations. The first is finding the coefficients of the characteristic polynomial in their highest order of J which we accomplished by implementing a set of symbolic rules in Mathematica. The second approximation is a distillation of the $O(J^n)$ coefficients, where n is the highest order of J, into the minimal number of conditions such that the coefficients of the characteristic polynomial are greater than zero. We accomplished this by looking for terms that are consistent between the coefficients and requiring them to be greater than zero. Often, two or more coefficients included the same terms. Because we matched subsets of terms and then required them to be greater than zero, our necessary conditions are somewhat more stringent than may be required by the coefficients. For example if two conditions appear in one coefficient it is possible that one of those conditions is significantly greater than zero, allowing the other condition flexibility in meeting its stability criterion. Since our purpose was to facilitate an understanding of the system analytically rather than calculate the exact stability requirements we felt that the trade off of simplicity for more stringent criteria was warranted. Here we will list the coefficients of the characteristic polynomial in the highest order of J for easier verification of our stability conditions:

$$a_0 = J_{ei}(J_{ie}^{ampa} + J_{ie}^{nmda}) - J_{ii}(J_{ee}^{ampa} + J_{ee}^{nmda}), \tag{S31}$$

$$\begin{aligned} a_1 = {} & J_{ei}(J_{ie}^{ampa} + J_{ie}^{nmda})(\tau_{ee}^{ampa} + \tau_{ee}^{nmda} + \tau_{ii}) \\ & - J_{ii}(J_{ee}^{ampa} + J_{ee}^{nmda})(\tau_{ie}^{ampa} + \tau_{ie}^{nmda} + \tau_{ei}) \\ & + J_{ei}(J_{ie}^{ampa}\tau_{ie}^{nmda} + J_{ie}^{nmda}\tau_{ie}^{ampa}) \\ & - J_{ii}(J_{ee}^{ampa}\tau_{ee}^{nmda} + J_{ee}^{nmda}\tau_{ee}^{ampa}), \end{aligned} \tag{S32}$$

$$\begin{aligned} a_2 = {} & J_{ei}J_{ie}^{ampa}\big(\tau_{ii}(\tau_{ee}^{nmda} + \tau_{ee}^{ampa}) + \tau_{ee}^{nmda}\tau_{ee}^{ampa} \\ & + \tau_{ie}^{nmda}(\tau_{ii} + \tau_{ee}^{nmda} + \tau_{ee}^{ampa})\big) \\ & + J_{ei}J_{ie}^{nmda}\big(\tau_{ii}(\tau_{ee}^{nmda} + \tau_{ee}^{ampa}) + \tau_{ee}^{nmda}\tau_{ee}^{ampa} \\ & + \tau_{ie}^{ampa}(\tau_{ii} + \tau_{ee}^{nmda} + \tau_{ee}^{ampa})\big) \\ & - J_{ii}J_{ee}^{ampa}\big(\tau_{ei}(\tau_{ie}^{nmda} + \tau_{ie}^{ampa}) + \tau_{ie}^{nmda}\tau_{ie}^{ampa} \\ & + \tau_{ee}^{nmda}(\tau_{ei} + \tau_{ie}^{nmda} + \tau_{ie}^{ampa})\big) \\ & - J_{ii}J_{ee}^{nmda}\big(\tau_{ei}(\tau_{ie}^{nmda} + \tau_{ie}^{ampa}) + \tau_{ie}^{nmda}\tau_{ie}^{ampa} \\ & + \tau_{ee}^{ampa}(\tau_{ei} + \tau_{ie}^{nmda} + \tau_{ie}^{ampa})\big) \end{aligned}$$

$$\begin{aligned} a_3 = {} & J_{ei}(J_{ie}^{nmda}\tau_{ie}^{ampa} + J_{ie}^{ampa}\tau_{ie}^{nmda}) \\ & * (\tau_{ii}(\tau_{ee}^{nmda} + \tau_{ee}^{ampa}) + \tau_{ee}^{nmda}\tau_{ee}^{ampa}) \end{aligned}$$

$$\begin{aligned}
&+ J_{ei}(J_{ie}^{nmda} + J_{ie}^{ampa})\tau_{ii}\tau_{ee}^{nmda}\tau_{ee}^{ampa} \\
&- J_{ii}(J_{ee}^{nmda}\tau_{ee}^{ampa} + J_{ee}^{ampa}\tau_{ee}^{nmda}) \\
&* (\tau_{ei}(\tau_{ie}^{nmda} + \tau_{ie}^{ampa}) + \tau_{ie}^{nmda}\tau_{ie}^{ampa}) \\
&- J_{ii}(J_{ee}^{nmda} + J_{ee}^{ampa})\tau_{ei}\tau_{ie}^{nmda}\tau_{ie}^{ampa} \qquad \text{(S33)}
\end{aligned}$$

$$\begin{aligned}
a_4 &= J_{ei}(J_{ie}^{nmda}\tau_{ie}^{ampa} + J_{ie}^{ampa}\tau_{ie}^{nmda})\tau_{ii}\tau_{ee}^{nmda}\tau_{ee}^{ampa} \\
&- J_{ii}(J_{ee}^{nmda}\tau_{ee}^{ampa} + J_{ee}^{ampa}\tau_{ee}^{nmda})\tau_{ei}\tau_{ie}^{nmda}\tau_{ie}^{ampa} \qquad \text{(S34)}
\end{aligned}$$

$$\begin{aligned}
a_5 &= J_{ii}(\tau_e\tau_{ei}\tau_{ie}^{ampa}\tau_{ie}^{nmda}\tau_{ee}^{nmda} + \tau_{ee}^{ampa}(\tau_e\tau_{ei}\tau_{ie}^{nmda}\tau_{ee}^{nmda} \\
&+ \tau_{ie}^{ampa}(\tau_e\tau_{ie}^{nmda}\tau_{ee}^{nmda} + \tau_{ei}(\tau_{ie}^{nmda}\tau_{ee}^{nmda} \\
&+ \tau_e(\tau_{ie}^{nmda} + \tau_{ee}^{nmda}))))) \\
&- J_{ee}^{nmda}(\tau_i\tau_{ei}\tau_{ii}\tau_{ie}^{ampa}\tau_{ie}^{nmda} + \tau_{ee}^{ampa}(\tau_i\tau_{ei}\tau_{ii}\tau_{ie}^{nmda} \\
&+ \tau_{ie}^{ampa}(\tau_i\tau_{ii}\tau_{ie}^{nmda} + \tau_{ei}(\tau_i\tau_{ie}^{nmda} + \tau_{ii}(\tau_i + \tau_{ie}^{nmda}))))) \\
&- J_{ee}^{ampa}(\tau_i\tau_{ei}\tau_{ii}\tau_{ie}^{nmda}\tau_{ee}^{nmda} + \tau_{ie}^{ampa}(\tau_i\tau_{ii}\tau_{ie}^{nmda}\tau_{ee}^{nmda} \\
&+ \tau_{ei}(\tau_i\tau_{ie}^{nmda}\tau_{ee}^{nmda} + \tau_{ii}(\tau_{ie}^{nmda}\tau_{ee}^{nmda} \\
&+ \tau_i(\tau_{ie}^{nmda} + \tau_{ee}^{nmda}))))) \qquad \text{(S35)}
\end{aligned}$$

$$\begin{aligned}
a_6 &= J_{ii}\tau_e\tau_{ee}^{ampa}\tau_{ee}^{nmda} - J_{ee}^{ampa}\tau_i\tau_{ii}\tau_{ee}^{nmda} \\
&- J_{ee}^{nmda}\tau_i\tau_{ii}\tau_{ee}^{ampa} \qquad \text{(S36)}
\end{aligned}$$

$$a_7 = 1 \qquad \text{(S37)}$$

$$a_8 = 1 \qquad \text{(S38)}$$

7 Changing the Synaptic Time Constants

Our model as described is a special case in which there are two types of excitatory synapses, of which each is composed entirely of either AMPA or NMDA receptors. However, in the linear case, our balanced network can be shown to be equivalent to one with different combinations of AMPA and NMDA receptors at its synapses. In addition, we will show that when STD is added, the STD model we presented is a special case of a more general model where each synapse has one type of STD and a ratio of AMPA to NMDA receptors.

Without STD, we consider two synapses that are allowed to have different ratios of NMDA to AMPA receptors. We use q again for the fraction of synaptic strength produced by the NMDA receptors in a given synapse. Each synapse uses a different combination of the exact same synaptic filters, either S_{ee}^{ampa} and S_{ee}^{nmda} or S_{ie}^{ampa} and S_{ie}^{nmda}. All synapses see the same average firing rate of the excitatory population, R_e, and therefore no change in the equations for the synapses are required. The only change is in how the outputs of the different synaptic filters are summed. A reformulation of Eqs (2)-(3) gives,

$$\tau_e \frac{dR_e}{dt} = -R_e + J_{ee}^{A}\left((1-q_A)S_{ee}^{ampa} + q_A S_{ee}^{nmda}\right)$$

$$+ J_{ee}^{B}\left((1-q_B)S_{ee}^{ampa} + q_B S_{ee}^{nmda}\right) - J_{ei}S_{ei} + I(t), \tag{S39}$$

$$\tau_i \frac{dR_i}{dt} = -R_i + J_{ee}^{C}\left((1-q_C)S_{ee}^{ampa} + q_C S_{ee}^{nmda}\right) + J_{ee}^{D}\left((1-q_D)S_{ee}^{ampa} + q_D S_{ee}^{nmda}\right) - J_{ii}S_{ii}. \tag{S40}$$

All variables are the same as previously described except that there are now four types of synapses labeled $A - D$ which have associated NMDA percentages $q_A - q_D$. If we reorganize these equations we find that they are exactly the same as Eqs (2)-(3) where the synaptic strengths are now defined such that,

$$J_{ee}^{ampa} = (1-q_A)J_{ee}^{A} + (1-q_B)J_{ee}^{B}, \tag{S41}$$

$$J_{ee}^{nmda} = q_A J_{ee}^{A} + q_B J_{ee}^{B}, \tag{S42}$$

$$J_{ie}^{ampa} = (1-q_C)J_{ie}^{C} + (1-q_D)J_{ie}^{D}, \tag{S43}$$

$$J_{ie}^{nmda} = q_C J_{ie}^{C} + q_D J_{ie}^{D}. \tag{S44}$$

Therefore, the linear model without STD is equivalent to one where each synapse can have a different ratio of NMDA to AMPA receptors.

When STD is added to Eqs (S39)-(S40) the coefficients are no longer equivalent. Now the coefficients representing the synaptic strengths include STD and hence are not constant,

$$J_{ee}^{ampa} = (1-q_A)x_1 J_{ee}^{A} + (1-q_B)x_2 J_{ee}^{B}, \tag{S45}$$

$$J_{ee}^{nmda} = q_A x_1 J_{ee}^{A} + q_B x_2 J_{ee}^{B}, \tag{S46}$$

$$J_{ie}^{ampa} = (1-q_C)x_2 J_{ie}^{C} + (1-q_D)x_1 J_{ie}^{D}, \tag{S47}$$

$$J_{ie}^{nmda} = q_C x_2 J_{ie}^{C} + q_D x_1 J_{ie}^{D}. \tag{S48}$$

where x_1 and x_2 represent the STD equations defined for the balanced network with STD. This new model will produce similar responses to the model with pure AMPA or NMDA synapses. When $q_A \to 0$, $q_B \to 1$, $q_C \to 0$ and $q_D \to 1$, Eqs (S45)-(S48) are equivalent to the model with STD presented in the main paper.

8 Steady States of the Network with STD

Since STD adds nonlinearities to the network, alterations in parameters or the strength of input do not necessarily yield easily predictable results. Therefore, in this section we analytically calculate the steady state values of the balanced network with STD, Eqs (2)-(4) and (9)-(12). Setting the derivatives equal to zero, assuming $I(t) = I$ is constant and solving for R_e yields a cubic polynomial. Its coefficients are,

$$a_0 = \frac{-I}{\tau_r^{ampa} u^{ampa} \tau_r^{nmda} u^{nmda}} \tag{S49}$$

$$a_1 = -I\left(\frac{1}{\tau_r^{ampa} u^{ampa}} + \frac{1}{\tau_r^{nmda} u^{nmda}}\right) + \frac{1 + \frac{kw^2}{1+kw} - w}{\tau_r^{ampa} u^{ampa} \tau_r^{nmda} u^{nmda}} \tag{S50}$$

$$a_2 = -I + \frac{1 + \frac{1}{2}\frac{kw^2}{1+kw} - \frac{1}{2}w}{\tau_r^{nmda} u^{nmda}} + \frac{1 + \frac{1}{2}\frac{kw^2}{1+kw} - \frac{1}{2}w}{\tau_r^{ampa} u^{ampa}} \tag{S51}$$

$$a_3 = 1. \tag{S52}$$

Each subscript corresponds to the respective power of R_e in the polynomial. We will refer to steady state values of R_e as R_e^{ss}. Given that we only consider situations where $I > 0$ then the coefficient a_0 must always be less than zero. Since coefficient a_3 is positive there is at least one change of signs in the coefficients. Thus, Descartes' rule of signs implies that there will always be at least one positive root for non-oscillatory solutions. Therefore, the number of positive and negative roots will be determined by coefficients a_1 and a_2. The right hand side of each of these equations is similar to the balance equation implying that both a_1 and a_2 will tend to switch signs together maintaining the single sign change within the coefficients. Consequently, there will generally be one positive root and two negative roots when $I > 0$ and the balance condition is met. Another way of saying this is that the firing rate of the excitatory population has only one steady state solution. This reduces finding the steady state of the system to just finding the largest root of the polynomial.

When the stimulus is removed such that $I = 0$ then $a_0 = 0$ giving a zero root. Therefore, as long as there are no positive roots, the system will always decay to zero. If $I = 0$, the system will have one sign change and consequently one positive root when a_1 and a_2 are negative. This occurs when positive feedback is greater than negative feedback as determined by the balance equation. Therefore, when recurrent excitation is large compared to the negative feedback the system will decay to a non-negative steady state. This analysis shows that the steady states of this system act exactly as we would expect based on the linear portion of the network. A constant input causes the system to move to one positive steady state. Removing the stimulus causes the system to decay to zero unless there is unbalanced positive feedback. We will not list steady state solutions for the other dependent variables but their steady state values can be easily calculated from R_e^{ss}.

9 Stability, rise time and oscillations

In the main text we predominantly used one set of values for the synaptic strength, $k = 1.2$ and $w = 30$. Supplementary **Fig S1A-E** shows how the network responds to changes in both variables. Panels **A**, **B** and **C** show how w and k impact instability, delta oscillations and τ_n for negative Δq. Panels **D** and **E** show how w and k impact instability and τ_n for positive Δq. Panel **E** is the rise time of each network for a particular Δq. Since the slope of the rise time as a function of Δq is close to linear, see **Fig 2C**, **Fig S1E** effectively represents the slope of that line and therefore the rate of change of τ_n as a function of Δq. The rate of change of τ_n is not equivalent to the maximum possible value of τ_n since networks with a smaller slope are also stable for a larger range of Δq, see **Fig S1D,E**. From **Fig S1A-E** we see that increasing the overall synaptic strength w makes the approach to both instability and oscillations occur for much smaller values of Δq. It also increases the rate of change of τ_n. On the other hand, increasing the ratio of inhibitory to excitatory strength, k, results in smaller τ_n across all values of Δq, **Fig S1C,E**. It also increases the range of Δq for which the balanced network is stable. For large values of k and small values of w the network is stable for all possible positive values of Δq. In **Fig S1D** this occurs when $\Delta q = 0.7$ since all activity on the EE projection is carried by NMDA receptors at this value of Δq. Although changing w and k does cause some some changes in the quantitative response characteristics of the network the overall qualitative response remains the same.

The resonant frequency at the AMPA instability is highly robust to changes in k and w with a total change of approximately 1.4 Hz for a wide range of synaptic strengths, **Fig S1F**. However, q and k are not the only parameters that can modify the resonant frequency. Alterations in the effective synaptic time constants on each projection or changes in the effective membrane time constants for individual neurons might produce larger changes in the resonant frequency. Changes of the effective synaptic time constant on the excitatory projections are equivalent to changing q, the proportion of synaptic strength through NMDA receptors. Additionally, we define f^τ as a fractional decrease in the membrane time constant such that $\tau_e^{new} = f^\tau \tau_e$ and $\tau_i^{new} = f^\tau \tau_i$. The parameter f^τ represents an effectively decreasing membrane time constant as would be observed for high levels of afferent or recurrent activity in a conductance based model [4]. **Fig S1G** shows the resonant frequency as a function of q and f^τ with $k = 1.2$ and $w = 30$. For reference, in the main text $q = 0.3$ and $f^\tau = 1.0$. The network still oscillates in the delta range for many values of q and f^τ. However, for $q < 0.3$ and $f^\tau < 0.3$ the resonant frequency enters the theta range and then the alpha range. If $k > 1.2$ the frequencies could even be slightly higher. Although shifts out of the delta range are possible, for the largest part of the parameter space oscillations remain within the delta range.

Fig S1H shows the evolution of the network's poles as a function of Δq. The imaginary axis is plotted in units of Hertz. When poles separate from the real axis and have a non-zero imaginary part, they cause oscillations in the network at a frequency equivalent to their imaginary part. Poles to the right of the imaginary axis (vertical solid black line) cause the network to become unstable. Four of the network's eight poles are visible in the plot. The other four poles are not relevant to our analysis. The right plot is a close up of the poles in the black box in the left plot. The two poles close to the real axis control both the AMPA dominated instability and the increase of τ_n. As Δq becomes increasingly negative, the two poles, visible in the right plot, separate from the real axis then approach and cross the imaginary axis as the system becomes unstable. This is what causes the delta oscillations and the subsequent instability. In the other direction, as Δq becomes large and positive, both poles move to the real line. One pole then moves off to infinity and the other pole approaches the imaginary axis again. As the second pole approaches the imaginary axis it increases τ_n. Although this pole gets very close to instability it never crosses the axis even as Δq becomes large. The instability at large Δq is due to the two poles visible in the 60 Hz range in the left plot. As Δq becomes larger they approach and then cross the imaginary axis. This is what causes the oscillatory instability in the gamma range.

References

1. Brunel N, Wang XJ. What determines the frequency of fast network oscillations with irregular neural discharges? I. Synaptic dynamics and excitation-inhibition balance. Journal of Neurophysiology. 2003;90(1):415–430.

2. Murphy BK, Miller KD. Balanced Amplification: A New Mechanism of Selective Amplification of Neural Activity Patterns. Neuron. 2009;61(4):635–648.

3. Lim S, Goldman MS. Balanced cortical microcircuitry for maintaining information in working memory. Nature neuroscience. 2013;16(9):1306–1314.

4. Bernander O, Douglas RD, Martin KAC, Koch C. Synaptic background activity influences spatiotemporal integration in single pyramidal cells. Proc Nat Acad Sci, USA. 1991;88:11569–11573.

Fig S1. Stability, rise time and oscillatory activity of the rate based model as a function of the network parameters. All networks use $q = 0.30$ unless otherwise noted. The white crosses on A-F represent the values of k and w used for the rate based network without STD in the main text. A: Change in q on the EE projection required to reach the AMPA dominated instability. The colorbar refers to negative values of Δq. B: Change in q on the EE projection required to reach the bifurcation yielding delta oscillations. The colorbar refers to negative values of Δq. C: The rise time in seconds at which the network begins to produce delta oscillations. D: Change in q on the EE projection required to reach the NMDA dominated instability. E: Rise time of the network for a constant value of Δq. Shows the slope of the rise time as a function of k and w. $\Delta q = 0.075$ was chosen to ensure that all instantiations of the network were stable and had minimal oscillations. F: Location of the peak in the frequency response as the network approaches the AMPA dominated instability. G: Location of the peak in the frequency response as the network approaches the AMPA dominated instability. Network parameters were $k = 1.2$ and $w = 30$. q is the proportion of synaptic strength through NMDA receptors. f^τ is a reduction in the membrane time constant of the excitatory and inhibitory neurons such that $\tau_e^{new} = f^\tau \tau_e$ and $\tau_i^{new} = f^\tau \tau_i$. H: Poles of the rate based network without STD plotted as a function of Δq. The imaginary axis is in units of Hz. The right panel is an expansion of the box in the left panel (black rectangle around the origin). Poles cross the imaginary axis for large positive Δq at about 60 Hz, corresponding to an oscillatory instability in the gamma range (blue circles, left panel), and for small negative Δq at about 2 Hz, corresponding to an oscillatory instability in the delta range (red circles, right panel).
